# Dppa2 and Dppa4 directly regulate the Dux-driven zygotic transcriptional program

**DOI:** 10.1101/gad.321174.118

**Published:** 2019-02-01

**Authors:** Mélanie Eckersley-Maslin, Celia Alda-Catalinas, Marloes Blotenburg, Elisa Kreibich, Christel Krueger, Wolf Reik

**Affiliations:** 1 Epigenetics Programme, Babraham Institute, Cambridge CB22 3AT, United Kingdom;; 2 Wellcome Trust Sanger Institute, Hinxton, Cambridge CB10 1SA, United Kingdom

**Keywords:** 2C-like cells, DNA methylation, Dppa2, Dppa4, Dux, Zscan4, embryonic stem cell, epigenetic, zygotic genome activation

## Abstract

The molecular regulation of zygotic genome activation (ZGA) in mammals remains an exciting area of research. Primed mouse embryonic stem cells contain a rare subset of “2C-like” cells that are epigenetically and transcriptionally similar to the two-cell embryo and thus represent an in vitro approximation for studying ZGA transcription regulation. Recently, the transcription factor Dux, expressed in the minor wave of ZGA, was described to activate many downstream ZGA transcripts. However, it remains unknown what upstream maternal factors initiate ZGA in either a Dux-dependent or Dux-independent manner. Here we performed a candidate-based overexpression screen, identifying, among others, developmental pluripotency-associated 2 (Dppa2) and Dppa4 as positive regulators of 2C-like cells and transcription of ZGA genes. In the germline, promoter DNA demethylation coincides with expression of Dppa2 and Dppa4, which remain expressed until embryonic day 7.5 (E7.5), when their promoters are remethylated. Furthermore, Dppa2 and Dppa4 are also expressed during induced pluripotent stem cell (iPSC) reprogramming at the time that 2C-like transcription transiently peaks. Through a combination of overexpression, knockdown, knockout, and rescue experiments together with transcriptional analyses, we show that Dppa2 and Dppa4 directly regulate the 2C-like cell population and associated transcripts, including Dux and the Zscan4 cluster. Importantly, we teased apart the molecular hierarchy in which the 2C-like transcriptional program is initiated and stabilized. Dppa2 and Dppa4 require Dux to initiate 2C-like transcription, suggesting that they act upstream by directly regulating Dux. Supporting this, ChIP-seq (chromatin immunoprecipitation [ChIP] combined with high-throughput sequencing) analysis revealed that Dppa2 and Dppa4 bind to the Dux promoter and gene body and drive its expression. Zscan4c is also able to induce 2C-like cells in wild-type cells but, in contrast to Dux, can no longer do so in Dppa2/4 double-knockout cells, suggesting that it may act to stabilize rather than drive the transcriptional network. Our findings suggest a model in which Dppa2/4 binding to the Dux promoter leads to Dux up-regulation and activation of the 2C-like transcriptional program, which is subsequently reinforced by Zscan4c.

## Abstract (precis)

In this study, Eckersley-Maslin et al. investigated the upstream maternal factors that initiate zygotic genome activation (ZGA) in either a Dux-dependent (a transcription factor expressed in the minor wave of ZGA) or Dux-independent manner. They performed a candidate-based overexpression screen, identifying developmental pluripotency-associated 2 (Dppa2) and Dppa4 as positive regulators of 2C-like cells and transcription of ZGA genes, and their results suggest that Dppa2/4 binding to the Dux promoter leads to Dux up-regulation and activation of the 2C-like transcriptional program, which is subsequently reinforced by Zscan4c.

Activation of transcription from the embryonic zygotic genome is a key concerted molecular and developmental event occurring in two waves at the one- to two-cell stage in mice and the four- to eight-cell stage in humans (for reviews, see Li et al. 2013; Jukam et al. 2017; Eckersley-Maslin et al. 2018; Svoboda 2018). Despite its importance, the precise molecular regulation of zygotic genome activation (ZGA) remains poorly understood. In particular, we still know little of the transcription factors and chromatin regulators that drive ZGA transcription and of their coordination. Recently, the transcription factor Dux was shown to bind and activate many such ZGA transcripts in an embryonic stem cell (ESC) model of ZGA and be required for correct preimplantation development (De Iaco et al. 2017; Hendrickson et al. 2017; Whiddon et al. 2017). However, Dux itself is expressed in only the first or minor wave of ZGA, and what regulates Dux remains unknown.

Mouse ESCs represent an ideal system to study the molecular mechanism governing ZGA. Under serum or primed culture conditions, ESCs are heterogeneous and contain a small percentage of cells that not only transiently express ZGA transcripts but also share certain epigenetic characteristics with the two-cell embryo (for reviews, see Ishiuchi and Torres-Padilla 2013; Eckersley-Maslin et al. 2018). These so-called “2C-like” ESCs can be easily identified using fluorescent reporters driven by the promoters of ZGA transcripts, such as the endogenous retrovirus MERVL or Zscan4 cluster (Zalzman et al. 2010; Macfarlan et al. 2012; Ishiuchi et al. 2015; Eckersley-Maslin et al. 2016). To date, repressors of the 2C-like state and ZGA gene transcription have been identified, including Kap1/Trim28 (Rowe et al. 2010; Macfarlan et al. 2011), the histone demethylase Lsd1/Kdm1a (Macfarlan et al. 2012), the histone chaperone Caf-1 (Ishiuchi et al. 2015), and the LINE1–nucleolin complex (Percharde et al. 2018), among others (Hisada et al. 2012; Maksakova et al. 2013; Schoorlemmer et al. 2014; Storm et al. 2014; Choi et al. 2017; Rodriguez-Terrones et al. 2017). However, aside from Dux, positive regulators that activate ZGA transcripts remain elusive.

Developmental pluripotency-associated 2 (Dppa2) and Dppa4 are small putative DNA-binding proteins expressed exclusively in preimplantation embryos, pluripotent cells, and the germline (Bortvin et al. 2003; Maldonado-Saldivia et al. 2007; Madan et al. 2009). These small proteins contain a DNA-binding SAP domain and a conserved histone-binding C-terminal domain (Maldonado-Saldivia et al. 2007; Masaki et al. 2010) and physically interact and localize to euchromatin (Masaki et al. 2007; Nakamura et al. 2011). Both single- and double-knockout ESCs retain expression of pluripotency markers and self-renewal (Madan et al. 2009; Nakamura et al. 2011), suggesting that these proteins are dispensable for stem cell pluripotency. Intriguingly, both single- and double-knockout mice survive early embryonic development only to develop lung and skeletal defects and perinatal lethality at a time when these genes are no longer expressed (Madan et al. 2009; Nakamura et al. 2011). This has led to suggestions that the proteins may be involved in epigenetic priming in early development; however, a role in preimplantation development or in regulating ZGA transcription has not been investigated.

In order to identify new positive regulators of ZGA transcription, we performed a screen in ESCs, identifying 12 chromatin and epigenetic factors that increase the percentage of 2C-like cells within a population. Among these were Dppa2 and Dppa4. We investigated the regulation of these two proteins, revealing that promoter DNA demethylation during the germline cycle coincides with their expression in vivo, including in the oocyte. Knockdown of either Dppa2 or Dppa4 reduces 2C-like cells as well as expression of ZGA transcripts. Furthermore, knockout of Dppa2 and/or Dppa4 is sufficient to completely abolish this cell population. Importantly, this phenotype can be restored upon re-expression of both Dppa2 and Dppa4 but not Zscan4c, confirming that these two proteins are necessary to activate expression of ZGA transcripts. Furthermore, we show that both Dppa2 and Dppa4 bind and activate Dux. Notably, Dux is required for Dppa2 and Dppa4 to activate the 2C-like state and ZGA transcription. Therefore, Dppa2 and Dppa4 act as master activators of a ZGA transcriptional program by directly regulating the ZGA transcription factor Dux.

## Results

### Candidate-based screen for epigenetic and chromatin regulators of ZGA using 2C-like ESCs

In mice, ZGA takes place in two waves: a minor wave that occurs predominantly at the paternal pronucleus in the zygote and a more substantial major wave that takes place in the two-cell embryo. Unfortunately, these stages of development are not easily manipulated on the scale required for high-throughput screens. To circumvent this, we took advantage of a spontaneously occurring rare subpopulation of primed mouse ESCs that express transcripts usually restricted to ZGA, including the MERVL endogenous retrovirus and Zscan4 cluster (Zalzman et al. 2010; Macfarlan et al. 2012; Ishiuchi et al. 2015; Eckersley-Maslin et al. 2016). These “2C-like” ESCs also share several epigenetic features with the two-cell embryo, including global DNA hypomethylation (Eckersley-Maslin et al. 2016; Dan et al. 2017), decondensed chromatin (Akiyama et al. 2015; Ishiuchi et al. 2015; Eckersley-Maslin et al. 2016), and increased histone mobility (Bošković et al. 2014).

**Figure 1.**
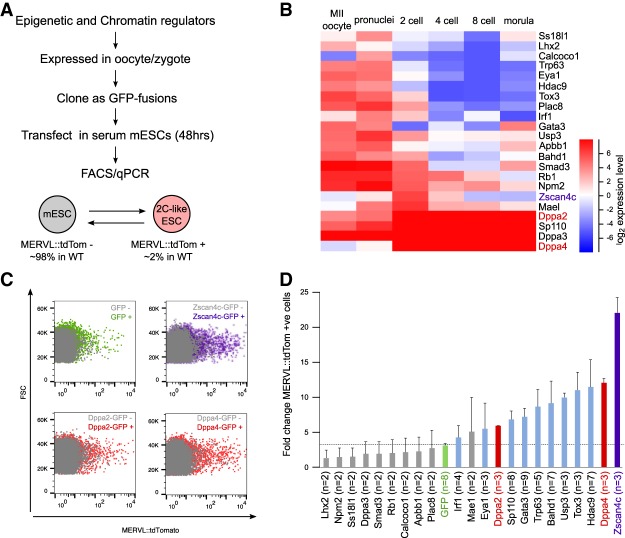
Screen for epigenetic and chromatin regulators of ZGA identifies Dppa2 and Dppa4 as potential regulators. (*A*) Overview of screen. Epigenetic and chromatin regulators expressed in oocytes and/or zygotes were cloned and transfected in serum ESCs for 48 h. Their ability to induce 2C-like transcription was measured by flow cytometry using the MERVL::tdTomato reporter and qRT–PCR on a panel of ZGA transcripts. (*B*) Heat map showing expression levels of factors screened in preimplantation embryos. Data are from Xue et al. (2013). (*C*) Representative flow cytometry plots showing levels of MERVL::tdTomato reporter (*X*-axis) following transfection of GFP (*top left*), Zscan4c-GFP (*top right*), Dppa2-GFP (*bottom left*), or Dppa4-GFP (*bottom right*) into ESCs. Untransfected cells are shown in gray, and transfected cells identified by GFP fluorescence are shown in the indicated color. (*D*) Expression of MERVL::tdTomato reporter following transfection of the corresponding GFP fusion constructs. The fold change between transfected GFP-positive cells over untransfected GFP-negative cells is shown. The GFP-only control is shown in green. Bars represent average plus standard deviation of at least two replicates. The number of replicates is denoted for each gene.

We first performed an in silico screen for potential positive regulators by selecting epigenetic and chromatin regulators that are expressed in the oocyte and/or zygote (Fig. 1A,B; see the Materials and Methods). As a positive control, we included Zscan4c, which has been implicated previously in activating early embryonic genes in stem cells (Hirata et al. 2012; Amano et al. 2013). Candidate genes were individually cloned as GFP fusions and transiently transfected into ESCs containing a tdTomato fluorescent reporter driven by the MERVL promoter (Fig. 1A; Macfarlan et al. 2012; Eckersley-Maslin et al. 2016). The ability of the individual genes to promote an early embryonic gene signature was tested both by flow cytometry analysis of the MERVL::tdTomato reporter (Fig. 1C,D) and quantitative RT–PCR (qRT–PCR) of a panel of ZGA transcripts (Supplemental Fig. 1A). Of the 22 candidates investigated, 12 promoted a 2C-like state by both flow cytometry and qRT–PCR. Following Zscan4c, Dppa4 was the strongest-scoring screen candidate, with its closely related and interacting partner, Dppa2, also among the screen hits, leading us to investigate these two genes further. Importantly, analysis of an independent microarray data set (Nishiyama et al. 2009) in which the transcriptome of ESCs following transcription factor overexpression was assessed revealed that just two of the 50 factors investigated promoted an early embryonic transcriptome (Supplemental Fig. 1B). Of the two factors that did promote expression of ZGA transcripts, Gata3 was similarly identified in our candidate-based screen, indicating that our bioinformatic preselection of candidates enriched substantially for potential ZGA regulators.

### Dppa2 and Dppa4 activate an early zygotic transcriptional network

**Figure 2.**
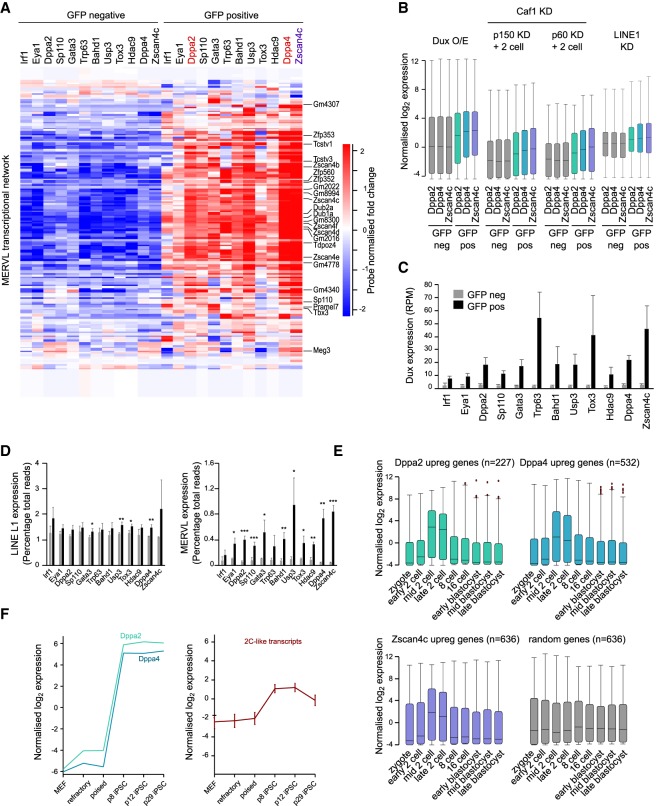
Transcriptome analysis reveals that Dppa2 and Dppa4 induce transcription of ZGA genes. (*A*) Heat map showing per-probe-normalized expression levels of ZGA transcripts expressed in 2C-like ESCs in GFP-negative (*left* set of columns) and transfected GFP-positive (*right* set of columns) sorted cells as measured by RNA-seq (three biological replicates per sample). The gene list is from Eckersley-Maslin et al. (2016). (*B*) Box and whisker plots showing expression of genes up-regulated by Dux overexpression (O/E) (data were reanalyzed from Hendrickson et al. 2017), Caf-1 p150 or p60 subunit knockdown (KD) and expressed in two-cell embryos (gene lists are from Ishiuchi et al. 2015), or LINE1 knockdown (gene list is from Percharde et al. 2018) in GFP-negative (gray) and GFP-positive (colored) cells following transfection of Dppa2-GFP (green), Dppa4-GFP (blue), or Zscan4c-GFP (purple). (*C*) Expression levels in RPM of the transcription factor Dux determined by RNA-seq in GFP-negative sorted (gray) and GFP-positive sorted (black) cells following transfection of the corresponding GFP-tagged constructs denoted *below* each pair of bars. Bars represent average plus standard deviation of three biological replicates. (*D*) Expression levels of LINE L1 elements (*left*) and MERVL elements (*right*) determined by RNA-seq in GFP-negative sorted (gray) and GFP-positive sorted (black) cells following transfection of the corresponding GFP-tagged constructs denoted *below* each pair of bars. Bars represent average plus standard deviation of at least three biological replicates. Differences are statistically significant. (*) *P*-value < 0.05; (**) *P*-value < 0.01; (***) *P*-value < 0.001, two-tailed homoscedastic *t*-test. (*E*) Expression patterns during preimplantation development of genes up-regulated by Dppa2 (green), Dppa4 (blue), Zscan4c (purple), or a random set of genes (gray). Preimplantation data are from Deng et al. (2014). (*F*) Expression patterns of Dppa2 and Dppa4 (*left*) and 2C-like transcripts (*right*) during induced pluripotent stem cell (iPSC) reprogramming. Data are reanalyzed from Milagre et al. (2017). (MEF) Mouse embryonic fibroblasts. Refractory (SSEA1^−^/Thy1^+^) and poised (SSEA1^+^/Thy1) stages correspond to fluorescence-activated cell sorting (FACS)-sorted cells at day 6, where passage 8 (p8; corresponding to day 21) and passage 12 (p12; corresponding to day 29) iPSCs represent intermediate–late stages of reprogramming, and passage 29 (p29; corresponding to day 60) iPSCs are fully reprogrammed.

To validate the 12 screen hits, we performed RNA sequencing (RNA-seq) of the GFP-positive and GFP-negative sorted cells following transient transfection of the relevant GFP fusion construct. Transcriptome analysis confirmed an up-regulation of 2C-like transcripts (Eckersley-Maslin et al. 2016) in the GFP-positive sorted cells compared with GFP-negative sorted controls (Fig. 2A; Supplemental Tables 1, 2). Consistently, the 12 screen hits also up-regulated genes that are similarly up-regulated following Dux overexpression (Hendrickson et al. 2017), Caf-1 knockdown (Ishiuchi et al. 2015), or LINE1 knockdown (Fig. 2B; Supplemental Fig. 2A; Percharde et al. 2018), indicating that the up-regulation of 2C-like transcripts is independent of how they are defined. To accurately determine transcript levels of Dux, we remapped the RNA-seq data to the Dux repeat sequence (see the Materials and Methods). Importantly, all 12 screen hits, including Zscan4c, Dppa2, and Dppa4, resulted in a significant up-regulation of Dux transcript (Fig. 2C). Interestingly, overexpression of Dux using a docycycline-inducible transgene induced expression of several of the screen hits, including the 2C-like genes Zscan4c and Sp110 as well as Dppa2 but not Dppa4 (Supplemental Fig. 2B). As all three of these genes are also up-regulated in 2C-like ESCs (Supplemental Fig. 2C), this could suggest that positive feedback loops act to reinforce the 2C-like state. Supporting this, analysis of published ChIP-seq (chromatin immunoprecipitation [ChIP] combined with high-throughput sequencing) data (Hendrickson et al. 2017) revealed Dux binding to the Dppa2 promoter (Supplemental Fig. 2D).

One of the hallmarks of 2C-like ESCs and preimplantation embryos is the up-regulation of repetitive elements, including the MERVL endogenous retrovirus (Macfarlan et al. 2012; Akiyama et al. 2015; Ishiuchi et al. 2015; Eckersley-Maslin et al. 2016). We therefore examined the repetitive portion of the transcriptome. Sequencing data were remapped to the consensus sequence of specific repeat families implicated in early embryonic development (see the Materials and Methods). There was a mild up-regulation of LINE L1 elements and a large up-regulation of MERVL elements in the GFP-positive sorted cells compared with GFP-negative sorted controls (Fig. 2D). Dppa2 and Dppa4 overexpression was able to induce an eightfold and 14-fold increase in MERVL expression, respectively, consistent with the MERVL::tdTomato reporter activation in these cells. Other repeat families, including IAP, MaLR, and major satellites, remained unchanged (Supplemental Fig. 2E), illustrating the specificity of repeat up-regulation.

One-hundred-ninety-five genes, including the 2C-like transcripts, were similarly up-regulated by Dppa2, Dppa4, and Zscan4c (Supplemental Fig. 2F). In all cases, the transcripts up-regulated by Dppa2, Dppa4, Zscan4c, or other screen hits, including Bahd1, Eya1, Hdac9, and Sp110, were similarly up-regulated in the mid to late two-cell stage during embryogenesis (Fig. 2E; Supplemental Fig. 2G), confirming that these transcripts are activated during ZGA. Other screen candidates, such as Gata3, Irf1, Tox3, and Trp63, up-regulated not only 2C-like transcripts but also other non-ZGA transcriptional networks (Supplemental Fig. 2G; Supplemental Table 2).

To further support the role of Dppa2 and Dppa4 in promoting expression of 2C-like ESCs, we looked at their expression patterns during induced pluripotent stem cell (iPSC) reprogramming. Up-regulation of 2C-like transcripts at intermediate stages of iPSC reprogramming has been reported previously (Eckersley-Maslin et al. 2016; Zhao et al. 2018). Consistently, both Dppa2 and Dppa4 are expressed when the ZGA transcripts are up-regulated (Fig. 2F). In summary, Dppa2 and Dppa4 up-regulate an early embryonic transcriptional program.

### Early embryonic and germline expression of Dppa2 and Dppa4 is regulated by promoter DNA demethylation

**Figure 3.**
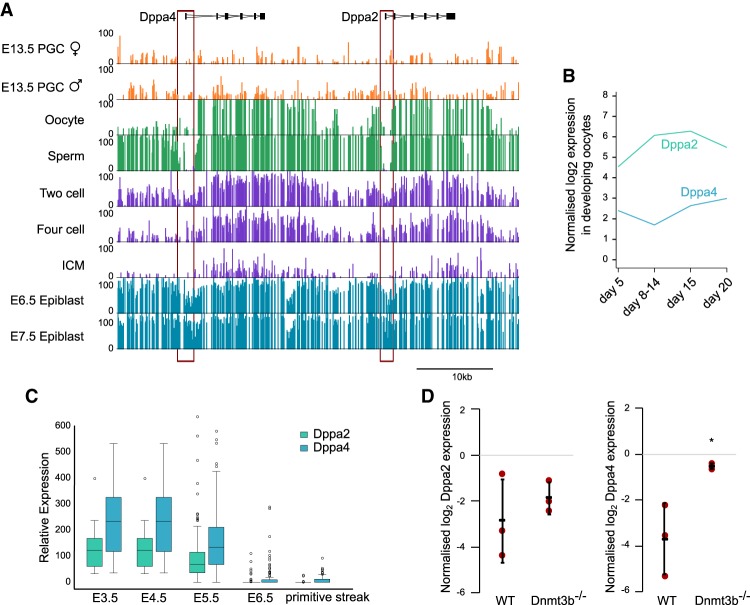
Expression of Dppa2 and Dppa4 coincides with promoter DNA hypomethylation. (*A*) Whole-genome bisulfite data showing the percentage of DNA methylation across the Dppa2/4 locus in male and female PGCs (orange), sperm and oocytes (green), two-cell and four-cell embryos, and inner cell mass (ICM) of blastocysts (purple), and E6.5 and E7.5 epiblast (blue). Gene structures are shown *above* tracks, and the approximate positions of DMRs are outlined in red boxes. Data were reanalyzed from Wang et al. (2014), except oocyte data, which are from Maenohara et al. (2017). (*B*) Expression levels of Dppa2 (green) and Dppa4 (blue) in nongrowing oocytes (postpartum day 5), growing oocytes (postpartum days 8–14 and 15), and fully-grown oocytes (postpartum day 20). Data were reanalyzed from Veselovska et al. (2015). (*C*) Expression levels of Dppa2 (green) and Dppa4 (blue) in single cells derived from E3.5, E4.5, E5.5, and E6.5 embryos and primitive streak. Data were reanalyzed from Mohammed et al. (2017). (*D*) Expression levels of Dppa2 (*left*) and Dppa4 (*right*) in wild-type (WT) and Dnmt3b^−/−^ embryos. Bars represent average ± standard deviation, and red dots indicate individual data points. (*) *P*-value < 0.05. Data were reanalyzed from Auclair et al. (2014).

Given the specific and restricted expression pattern of Dppa2 and Dppa4 in the germline and early embryo, we investigated the regulation of Dppa2 and Dppa4 in vivo. Primordial germ cells (PGCs) undergo a wave of DNA demethylation, which is then re-established in the mature gametes before a second wave of DNA demethylation takes place after fertilization in the preimplantation embryo (for reviews, see Lee et al. 2014; Eckersley-Maslin et al. 2018). Consistently, in both male and female PGCs, the *Dppa2/4* locus is demethylated (Fig. 3A; Supplemental Fig. 3A,B), which coincides with their expression in the gonads (Maldonado-Saldivia et al. 2007) and developing oocytes (Fig. 3B). In sperm and oocytes, there is a gain in DNA methylation across the locus; however, the promoters of both *Dppa2* and *Dppa4* remain hypomethylated (Fig. 3A; Supplemental Fig. 3A). This is in contrast to 2C-like gene promoters that are more highly methylated compared with all gene promoters in sperm (Supplemental Fig. 3C). During preimplantation, there is a second wave of DNA demethylation across the entire *Dppa2/4* locus (Fig. 3A). After implantation, levels of DNA methylation, including at the promoter, increase dramatically, consistent with the rapid silencing of Dppa2 and Dppa4 (Fig. 3C). The promoters of *Dppa2* and *Dppa4* remain methylated across all somatic tissues in which Dppa2 and Dppa4 are not expressed (Supplemental Fig. 3D). To further investigate the link between promoter DNA methylation and Dppa2/4 expression, we investigated transcriptome data from embryonic day 8.5 (E8.5) embryos that lacked the de novo DNA methyltransferase Dnmt3b, which is primarily responsible for establishing DNA methylation at promoter regions (Auclair et al. 2014). Importantly, there was an increase in both Dppa2 and Dppa4 expression in Dnmt3b^−/−^ embryos at a time when they are usually completely silenced (Fig. 3D), supporting a role for promoter DNA methylation in repressing these two genes in vivo. In summary, the *Dppa2* and *Dppa4* genes are primarily regulated by global demethylation during germline and early embryo development, and their products are therefore present in the oocyte at fertilization.

### Reducing levels of Dppa2 and Dppa4 leads to a reduction in 2C-like cells and Dux transcription

**Figure 4.**
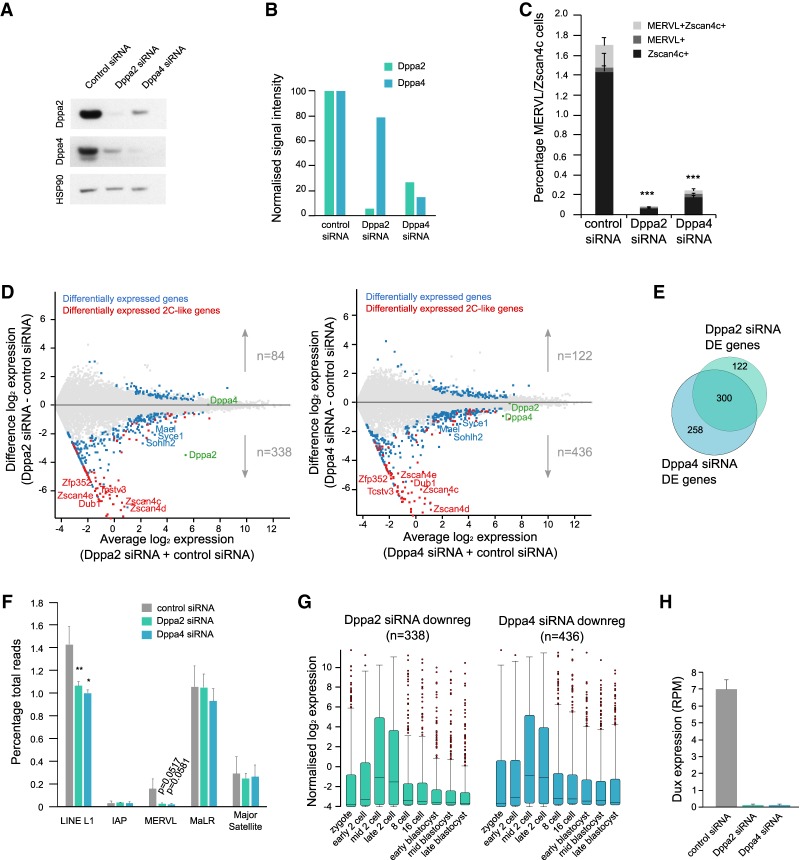
Knockdown of Dppa2 or Dppa4 reduces expression of ZGA transcripts. (*A*) Western blotting for Dppa2 (*top* row) and Dppa4 (*middle* row) following treatment with control (*left* column), Dppa2 (*middle* column), or Dppa4 (*right* column) siRNA. (*Bottom* row) HSP90 is shown as loading control. (*B*) Relative quantification of Western blotting normalized to HSP90 levels for each sample individually. (*C*) Flow cytometry analysis of reporter ESCs showing the percentage of 2C-like cells (Zscan4c^+^ and/or MERVL^+^) following treatment with either control or target siRNA. Error bars represent standard deviation of three to six biological replicates. Statistical analysis was done on total percentage of 2C-like cells (sum of Zscan4^+^, MERVL^+^, and Zscan4c^+^MERVL^+^ populations). (***) *P*-value < 0.001, two-tailed equal variance *t*-test. (*D*) MA plots showing average log_2_ expression versus difference in log_2_ expression for control siRNA and Dppa2 siRNA-treated (*left*) or Dppa4 siRNA-treated (*right*) ESCs analyzed by RNA-seq. Differentially expressed genes are highlighted in blue, and differentially expressed ZGA transcripts expressed in 2C-like ESCs are highlighted in red. Dppa2 and Dppa4 are indicated. (*E*) Overlap between differentially expressed (DE) genes following Dppa2 or Dppa4 siRNA treatment compared with control siRNA-treated cells. (*F*) Expression levels of various repeat classes in control siRNA-treated (gray), Dppa2 siRNA-treated (green), and Dppa4 siRNA-treated (blue) cells. Error bars represent average plus standard deviation of three biological replicates. Differences are statistically significant. (*) *P* < 0.05; (**) *P* < 0.01, two-tailed homoscedastic *t*-test. (*G*) Expression pattern of differentially down-regulated genes following Dppa2 siRNA treatment (green; *left*) or Dppa4 siRNA treatment (blue; *right*) during preimplantation development. Preimplantation data are from Deng et al. (2014). (*H*) Expression levels of Dux transcript in control siRNA-treated (gray), Dppa2 siRNA-treated (green), and Dppa4 siRNA-treated (blue) ESCs.

To test their necessity for ZGA transcripts expression, we first performed Dppa2 and Dppa4 knockdowns in MERVL::tdTomato/ Zscan4c::eGFP reporter serum ESCs (Eckersley-Maslin et al. 2016). Expression of either reporter accurately labels 2C-like cells (Zalzman et al. 2010; Macfarlan et al. 2012; Eckersley-Maslin et al. 2016). Cells were transfected with either control, Dppa2, or Dppa4 targeting siRNAs for 4 d, achieving 92% and 74% knockdown efficiency at the mRNA level, respectively (Supplemental Fig. 4A). This corresponded to 94% and 85% reduction in protein levels for Dppa2 and Dppa4, respectively (Fig. 4A,B). Interestingly, depletion of Dppa4 protein also reduced Dppa2 protein levels by 73% (Fig. 4A,B) despite mRNA levels remaining the same (Supplemental Fig. 4A), potentially due to protein destabilization. Dppa2 siRNA also led to a 22% reduction in Dppa4 protein levels (Fig. 4A,B). Analysis of the MERVL::tdTomato/ Zscan4c::eGFP reporters by flow cytometry revealed a dramatic depletion of the 2C-like ESC population (Fig. 4C), which was consistently reflected in the expression of selected ZGA transcripts, including Dux, by qRT–PCR, while pluripotency markers remained unchanged (Supplemental Fig. 4B). To further investigate the transcriptional changes occurring after Dppa2 or Dppa4 knockdown, we performed RNA-seq. The majority of differentially expressed transcripts were down-regulated, overlapped with 2C-like transcripts (Fig. 4D), and were similarly deregulated between Dppa2 and Dppa4 knockdowns (Fig. 4E; Supplemental Tables 3, 4). In addition to ZGA transcripts, there was milder down-regulation of a second group of genes in the knockdown samples that contained many lineage markers such as the gametogenesis genes Syce1, Sohlh2, and Mael (Fig. 4D), consistent with knockout ESC studies (Madan et al. 2009). Thus, while Dppa2 and Dppa4 likely have additional roles, the largest changes in gene expression occurred at the 2C-like transcripts. Analysis of the repetitive proportion of the genome revealed a down-regulation of LINE L1 and near absence of MERVL element expression (Fig. 4F). The down-regulated gene transcripts were expressed at the time of ZGA in preimplantation embryos (Fig. 4G). Transcripts expressed in 2C-like ESCs (Eckersley-Maslin et al. 2016) as well as those that are up-regulated following Dppa2, Dppa4, or Dux overexpression (Hendrickson et al. 2017) or following CAF-1 (Ishiuchi et al. 2015) or LINE1 knockdown (Percharde et al. 2018) were all down-regulated following Dppa2 or Dppa4 knockdown (Supplemental Fig. 4C), indicating that the same set of ZGA transcripts is being regulated by Dppa2 and/or Dppa4 irrespective of how they are defined. Importantly, transcript levels of the Dux transcription factor were barely detected following Dppa2 and Dppa4 knockdown (Fig. 4H). Therefore, Dppa2 and Dppa4 knockdown results in a decrease in Dux expression, 2C-like transcripts, and cells in the 2C-like state.

### Dppa2 and Dppa4 are both necessary for 2C-like transcript activation

**Figure 5.**
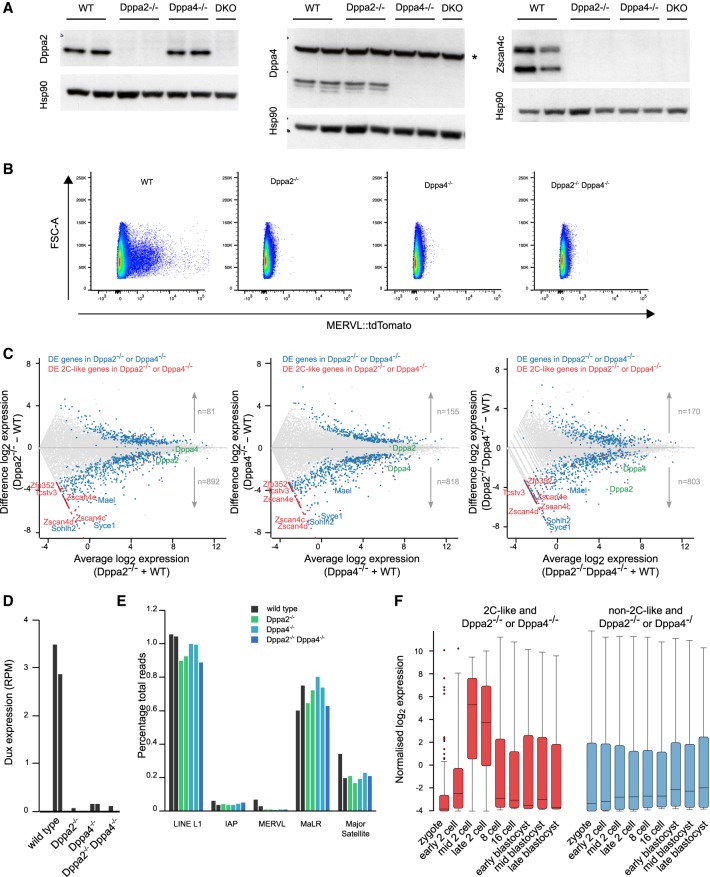
Knockout of Dppa2 and/or Dppa4 in ESCs abolishes 2C-like cells and ZGA transcript expression. (*A*) Western blotting for Dppa2 (*left*), Dppa4 (*middle*), and Zscan4c (*right*) in individual clones for wild-type (WT), Dppa2^−/−^, Dppa4^−/−^, and double Dppa2^−/−^ Dppa4^−/−^ (double knockout [DKO]) ESCs. Two clones are shown for wild type and single knockout, and one clone is shown for double knockout. Hsp90 was used as loading control. Note the presence of a nonspecific band (denoted by an asterisk) in the Dppa4 blot. (*B*) Flow cytometry plots showing expression of the MERVL::tdTomato reporter (*X*-axis) in wild-type (WT), Dppa2^−/−^, Dppa4^−/−^, and Dppa2^−/−^Dppa4^−/−^ double-knockout ESCs. (*C*) MA plots showing average log_2_ expression versus difference in log_2_ expression for wild-type (WT), Dppa2^−/−^ (*left*), Dppa4^−/−^ (*middle*), or Dppa2^−/−^Dppa4^−/−^ ESCs (*right*), analyzed by RNA-seq. Differentially expressed genes in either Dppa2^−/−^ or Dppa4^−/−^ ESCs are highlighted in blue, and differentially expressed ZGA transcripts expressed in 2C-like ESCs are highlighted in red. Dppa2 and Dppa4 are indicated. (*D*) Expression levels of Dux transcript in wild-type (WT), Dppa2^−/−^, Dppa4^−/−^, or Dppa2^−/−^Dppa4^−/−^ ESCs. (*E*) Expression levels of various repeat classes in wild-type (dark gray), Dppa2^−/−^ (green), Dppa4^−/−^ (light blue), and Dppa2^−/−^ Dppa4^−/−^ (dark blue) cells. (*F*) Expression patterns during preimplantation development of differentially expressed genes in either Dppa2^−/−^ or Dppa4^−/−^ ESCs and overlapping (red; *left*) or not overlapping (blue; *right*) 2C-like transcripts. Preimplantation data are from Deng et al. (2014).

To confirm that Dppa2 and Dppa4 are required for ZGA-like gene transcription, we generated single- and double-knockout ESCs deficient for Dppa2 and/or Dppa4 using CRISPR–Cas9 targeting in MERVL::tdTomato reporter cells. Knockout of either or both proteins was confirmed by Western blotting (Fig. 5A). Strikingly, flow cytometry analysis of the MERVL::tdTomato reporter revealed that both single- and double-knockout ESCs completely lacked the 2C-like subpopulation (Fig. 5B). Next, we performed RNA-seq to further investigate the transcriptional changes that occur following loss of Dppa2 and/or Dppa4 (see the Materials and Methods). We observed a dramatic depletion of 2C-like transcripts, including the Zscan4 cluster, Tcstv3, and Zfp352 (Fig. 5C; Supplemental Fig. 5A; Supplemental Tables 5, 6), consistent with the knockdown experiments. The absence of Zscan4c was validated at the protein level by Western blotting (Fig. 5A). Furthermore, expression of the ZGA transcription factor Dux (Fig. 5D) and MERVL endogenous retrovirus (Fig. 5E) was mostly absent in the knockout cells. Despite the loss of the 2C-like state, the cells retained mRNA and protein expression of the pluripotency markers Nanog and Oct4 (Supplemental Fig. 5A–C), grew at a similar rate, and were able to be passaged for >20 generations (data not shown), consistent with previous reports (Madan et al. 2009; Nakamura et al. 2011). Thus, these results indicate that the 2C-like state as well as Zscan4 protein expression are dispensable for sustained self-renewal in culture.

The intersection of differentially expressed genes from either Dppa2^−/−^ or Dppa4^−/−^ ESCs was similarly deregulated in the Dppa2^−/−^Dppa4^−/−^ ESCs (Fig. 5C; Supplemental Fig. 5D), suggesting that the three genotypes are largely indistinguishable transcriptionally. Similar to the knockdown experiments, we also observed a second group of misregulated genes in the knockout ESCs that included many lineage-specific genes, including Mael, Syce1, and Sohlh2, consistent with previous findings (Madan et al. 2009). However, unlike the differentially expressed 2C-like transcripts, this second group of differentially expressed genes is not normally up-regulated at the time of ZGA during preimplantation development (Fig. 5F) and therefore likely represents a separate function of Dppa2 and Dppa4 outside of regulating 2C-like transcription. Interestingly, there were significant transcriptional differences between siRNA knockdown and CRISPR knockout approaches (Supplemental Fig. 5E,F). While the majority of genes, including the 2C-like genes, Mael, Syce1, and Sohlh2, showed at least a trend toward down-regulation in both siRNA knockdown and CRISPR knockout, there were other genes that were up-regulated and down-regulated specifically in the knockout cells compared with siRNA knockdown and vice versa (Supplemental Fig. 5G–K). These genes may be sensitive to either the dosage of Dppa2/4 or the duration of Dppa2/4 depletion and thus may represent secondary adaptive effects of Dppa2/4 deletion. Despite these differences, the 2C-like genes were down-regulated by both approaches. Therefore, Dppa2 and Dppa4 are necessary for ZGA transcript expression in ESCs.

**Figure 6.**
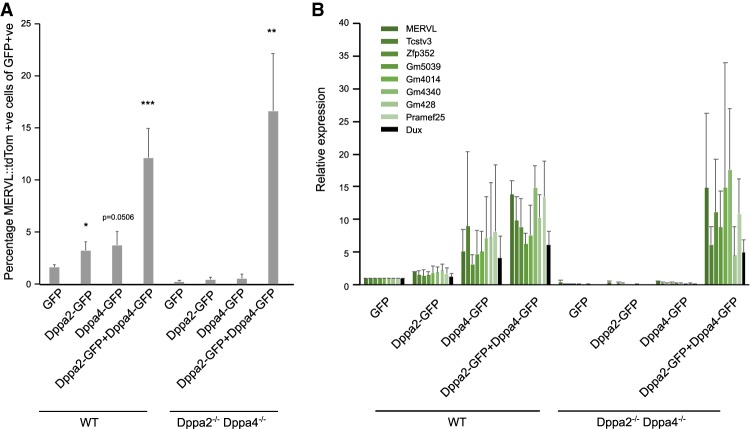
Rescue of Dppa2 and Dppa4 restores 2C-like cells and ZGA transcript expression. Rescue experiments in wild-type (WT; *left*) and Dppa2/4 double knockout (*right*) ESCs. Cells were transfected with GFP, Dppa2-GFP, Dppa4-GFP, or Dppa2-GFP with Dppa4-GFP constructs for 48 h. (*A*) Expression of the MERVL::tdTomato reporter as measured by flow cytometry. (*B*) Expression of ZGA transcripts as measured by qRT–PCR. Differences are statistically significant. (*) *P*-value < 0.5; (**) *P*-value < 0.01; (***) *P*-value < 0.001, homoscedastic two-tailed *t*-test. Error bars represent average plus standard deviation of three biological replicates.

Next, we performed rescue experiments in the double-knockout ESCs. Consistent with our initial screen, overexpression of Dppa4 and Dppa2 in wild-type cells (Supplemental Fig. 6A) up-regulated the 2C-like cell fraction by flow cytometry (Fig. 6A). Expression of 2C-like transcripts, including Dux (Fig. 6B), was increased. Overexpressing both Dppa2 and Dppa4 resulted in a larger up-regulation in 2C-like cells and associated transcripts than either one alone (Fig. 6A,B), consistent with them acting in a complex (Nakamura et al. 2011). Consistently, in Dppa2/Dppa4-null ESCs, Dppa2 was not able to induce the 2C-like state or associated transcripts, and Dppa4 alone led to only a modest increase. Moreover, overexpression of Zscan4c was not able to rescue the Dppa2/Dppa4 knockout phenotype (Supplemental Fig. 6B), suggesting that Zscan4c requires Dppa2 and Dppa4 to enhance the 2C-like cell state. Importantly, reintroduction of both Dppa2 and Dppa4 resulted in a substantial increase in the MERVL::tdTomato-positive cell fraction (Fig. 6A) and associated transcripts, including Dux (Fig. 6B). Therefore, Dppa2 and Dppa4 together drive the 2C-like cell state and ZGA-like transcriptional network.

### Dppa2 and Dppa4 directly bind and regulate the transcription factor Dux

**Figure 7.**
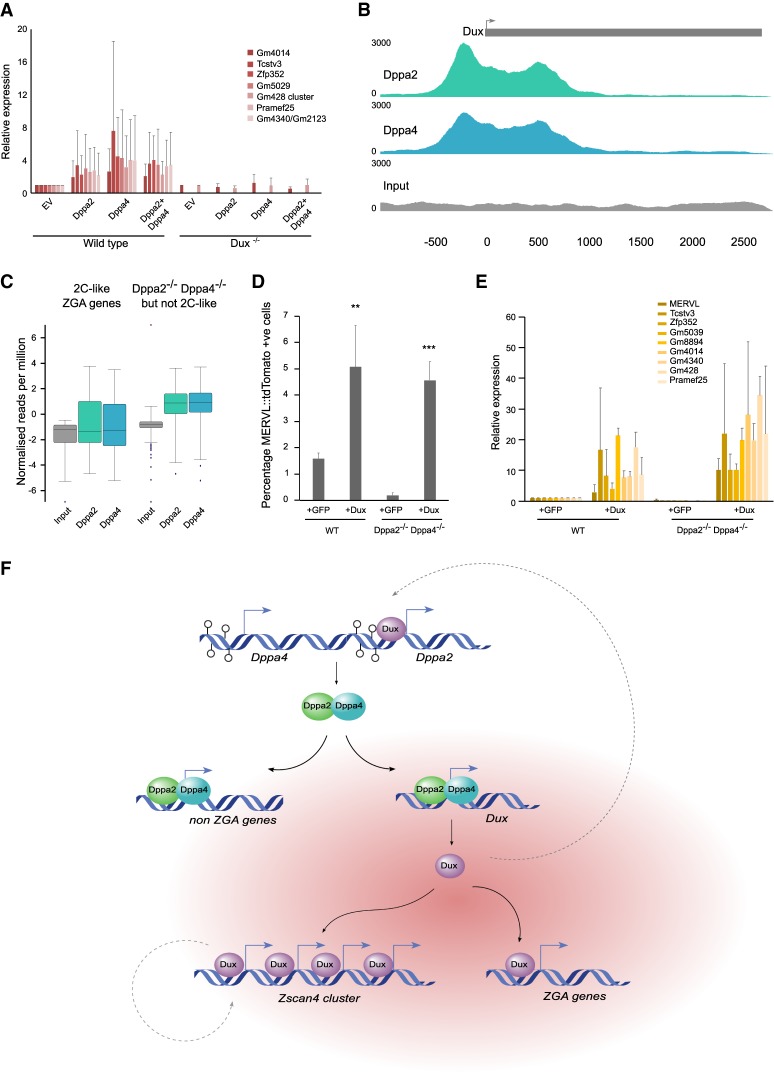
Dppa2 and Dppa4 bind and regulate Dux, which in turn is required to up-regulate ZGA transcripts. (*A*) qRT–PCR analysis of 2C-like transcripts following transient transfection of untagged Dppa2 and/or Dppa4 in wild-type (*left*) and Dux^−/−^ (*right*) ESCs, using transfection of an empty vector (EV) as a control. (*B*) ChIP-seq analysis of endogenous Dppa2 (green; *top*) and Dppa4 (blue; *middle*) binding to the Dux consensus sequence and promoter region. The Dux transcriptional unit is denoted in gray, and the scale represents base pairs relative to the transcriptional start site (TSS). (*Bottom* row) Input (gray) is shown. Data were reanalyzed from Hernandez et al. (2018). (*C*) Box and whisker plots showing enrichment of Dppa2 (green) and Dppa4 (blue) at differentially expressed genes following Dppa2 or Dppa4 knockdown that either overlap (*left*) or do not overlap (*right*) with 2C-like transcripts. Probes were made across TSSs (±500 base pairs [bp]), and normalized counts per million reads were determined and compared with control/input (gray). Data were reanalyzed from Hernandez et al. (2018). (*D*) Flow cytometry analysis of reporter ESCs showing the percentage of MERVL::tdTomato+ve 2C-like cells in wild-type (WT) or Dppa2^−/−^Dppa4^−/−^ ESCs transfected with Dux or GFP-positive sorted cells as a control. Error bars represent standard deviation of three biological replicates. Differences are statistically significant. (**) *P*-value < 0.01; (***) *P*-value < 0.001, two-tailed homoscedastic *t*-test. (*E*) qRT–PCR analysis of 2C-like transcripts in cells in wild-type or Dppa2^−/−^Dppa4^−/−^ ESCs transfected with Dux or GFP control. (*F*) Promoter DNA demethylation (open circles) enables expression of Dppa2 (green) and Dppa4 (blue), which bind to both non-ZGA genes and, under permissive conditions (red cloud), the ZGA transcription factor Dux, inducing its expression. Dux (purple) then binds and activates ZGA genes, including the Zscan4 cluster of genes. Dotted gray lines represent positive feedback loops.

Our results so far have revealed a role for Dppa2 and Dppa4 in regulating the 2C-like state and ZGA-like transcripts. Additionally, Dppa2 and Dppa4 are necessary and sufficient to regulate expression of the ZGA transcription factor Dux, which itself has been shown recently to regulate a similar ZGA transcriptional program (De Iaco et al. 2017; Hendrickson et al. 2017; Whiddon et al. 2017). To determine whether Dppa2 and Dppa4 act to regulate Dux directly or exert their effects through parallel pathways, we determined whether Dux is required for Dppa2 and Dppa4 to regulate the 2C-like transcripts. Wild-type and Dux knockout ESCs (De Iaco et al. 2017) were cultured in serum conditions and transfected with constructs containing Dppa2, Dppa4, or both Dppa2 and Dppa4 constructs simultaneously and compared with those receiving an empty vector (Supplemental Fig. 7A,B). While Dppa2 and/or Dppa4 were able to induce expression of the 2C-like transcripts in wild-type cells, this ability was abolished in Dux knockout cells (Fig. 7A). Therefore, Dux is required for the transcriptional effects exerted by Dppa2 and Dppa4.

Next, we analyzed published Dppa2 and Dppa4 ChIP-seq data (Engelen et al. 2015; Hernandez et al. 2018; Klein et al. 2018) to determine whether these proteins directly bind the Dux repeat (see the Materials and Methods). Importantly, we observed clear enrichment of Dppa2 and Dppa4 binding across the promoter and into the Dux transcript itself in E14 ESCs (Fig. 7B; Supplemental Fig. 7C). Furthermore, Dppa4 similarly bound to Dux in P19 embryonal carcinoma cells but not in 3T3 fibroblasts (Supplemental Fig. 7C). Thus, Dppa2 and Dppa4 directly bind to Dux in PSCs, consistent with when Dux is expressed. We next investigated whether Dppa2 and Dppa4 bind to other genes that are differentially expressed in Dppa2^−/−^ or Dppa4^−/−^ ESCs. There was a strong enrichment for both Dppa2 and Dppa4 at the promoters of the non-2C-like genes (Fig. 7C), including Syce1, Sohlh2, and Mael (Supplemental Fig. 7D–F), again confirming a separate role for Dppa2 and Dppa4 in regulating non-ZGA transcripts in ESCs. Importantly, Dppa2 and Dppa4 did not bind to the transcriptional start sites (TSSs) of other 2C-like transcripts (Fig. 7C), including the Zscan4 cluster, Gm428, and Dub1 (Supplemental Fig. 7G–I), which are direct targets of the Dux protein itself (Hendrickson et al. 2017).

Our results support a model in which Dppa2 and Dppa4 act by directly regulating levels of the Dux transcription factor, which in turns acts to bind and promote expression of a zygotic transcriptional program in ESCs. Therefore, expressing Dux in the absence of Dppa2 and Dppa4 should restore the 2C-like state and associated ZGA transcripts. To this end, we overexpressed Dux in the Dppa2^−/−^ Dppa4^−/−^ ESCs (Supplemental Fig. 7J). Indeed, flow cytometry analysis of the MERVL::tdTomato reporter revealed that Dux overexpression is able to induce the 2C-like state in both wild-type and Dppa2^−/−^Dppa4^−/−^ ESCs (Fig. 7D). Furthermore, expression of 2C-like transcripts was induced following Dux overexpression (Fig. 7E). Together, this suggests that Dux acts downstream from Dppa2 and Dppa4. In summary, Dppa2 and Dppa4 induce the 2C-like state and associated ZGA transcripts by directly binding and activating the ZGA transcription factor Dux, which is then able to bind and activate downstream 2C-like target genes.

## Discussion

Initiation of transcription of the zygotic genome is a critical step in embryogenesis. To understand its molecular regulation, we performed a screen for chromatin and epigenetic regulators of ZGA transcription using 2C-like ESCs as a model, identifying, among others, Dppa2 and Dppa4. Here, we propose a model in which promoter DNA demethylation during global epigenetic reprogramming enables expression of Dppa2 and Dppa4 in the germline and oocytes. Dppa2 and Dppa4 then directly bind and up-regulate both non-ZGA genes as well as, under permissive conditions such as chromatin decompaction, the ZGA transcription factor *Dux* (Fig. 7F). Dux is subsequently able to bind and activate downstream ZGA transcription, including the *Zscan4* cluster. Several feedback loops reinforce this system, including Zscan4-induced stabilization as well as Dux-induced up-regulation of *Dppa2*. Our study provides crucial insights into the molecular hierarchy that triggers ZGA transcription and links it with epigenetic reprogramming in the germline.

The existence of a 2C-like state in ESCs, while not the same as the two-cell embryo, represents a useful in vitro approximation for studying ZGA, making many molecular and screening-based studies possible. This state is characterized by an increase in chromatin mobility (Bošković et al. 2014), decondensed chromocenters (Akiyama et al. 2015; Ishiuchi et al. 2015), and increased chromatin accessibility (Eckersley-Maslin et al. 2016) and global DNA hypomethylation (Eckersley-Maslin et al. 2016; Dan et al. 2017). Consistently, depletion of factors involved in chromatin assembly (Ishiuchi et al. 2015) or treatment with inhibitors that ultimately induce chromatin decompaction (Macfarlan et al. 2012; Dan et al. 2015) increases the proportion of these cells in culture. Furthermore, knockdown or knockout of many repressive epigenetic regulators, including the histone demethylase Kdm1a (Macfarlan et al. 2011), histone methyltransferase Ehmt2 (Macfarlan et al. 2012), heterochromatin protein HP1 (Maksakova et al. 2013), and components of the PRC1.6 subcomplex (Rodriguez-Terrones et al. 2017) among others (Maksakova et al. 2013; Dan et al. 2014; Fujii et al. 2015; Rodriguez-Terrones et al. 2017), have also been shown to enhance the 2C-like state.

In PGCs, the promoters of *Dppa2* and *Dppa4* are demethylated, coinciding with their expression. They remain robustly expressed through preimplantation development, including at the time of ZGA, until the onset of gastrulation, when expression rapidly ceases and their promoters reacquire DNA methylation. Dppa2 is highly expressed in oocytes and zygotes prior to ZGA, and, while Dppa4 transcripts are less abundant, proteins for both Dppa2 and Dppa4 are readily detectable (Pfeiffer et al. 2011). Additionally, Dppa2 and Dppa4 are expressed at the time during iPSC reprogramming, when 2C-like transcripts are transiently expressed. It will be interesting to determine whether Dppa2/4-mediated passage through a 2C-like state is required for iPSC reprogramming. Importantly, Dppa2 and Dppa4 are homogeneously expressed across all ESCs, not just 2C-like cells (data reanalyzed from Eckersley-Maslin et al. 2016). However, this raises the question of why the ZGA genes are expressed in only a small subset of, but not all, ESCs. Activation of the 2C-like state is likely a multifaceted process requiring the presence of not only the upstream activators Dppa2 and Dppa4 but also chromatin decompaction and/or reduced expression of repressors such as Kap1 or PRC1 and their modifications. Once activated, factors such as Zscan4c may act to stabilize and prolong expression of the transcriptional program, which then requires repressors such as the LINE-1/Nucleolin complex (Percharde et al. 2018), NuRD, or Caf-1 (Ishiuchi et al. 2015; Campbell et al. 2018) to repress it once again. In this way, Dppa2 and Dppa4 regulate the entry into the 2C-like state during permissive conditions by directly activating the ZGA major transcription factor Dux, which subsequently activates the remainder of the ZGA transcripts in the cell.

In this study, we used Zscan4c as a positive control in the candidate-based screen. Zscan4c is one of a tandemly encoded family of zinc finger and SCAN domain-containing proteins that are expressed in early embryos and in 1%–5% of ESCs (Zalzman et al. 2010), including the rarer MERVL-positive 2C-like cells (Eckersley-Maslin et al. 2016; Rodriguez-Terrones et al. 2017). Zscan4c has been implicated as a positive regulator of 2C-like cells (Hirata et al. 2012; Amano et al. 2013), which we confirmed here. However, Zscan4c is unable to induce the 2C-like state or ZGA-like transcription in the absence of Dppa2, Dppa4, or Dux (data not shown). While it remains to be determined whether Zscan4c or other members of the Zscan4 cluster are necessary for ZGA transcription, our results suggest that Zscan4c may act to stabilize or reinforce the 2C-like state rather than induce it. Interestingly, as well as Zscan4c, Sp110 is also up-regulated in 2C-like cells and following Dux overexpression and was also identified in our screen as a positive regulator of the 2C-like state. It will be interesting to see whether it may also act as a reinforcer of the 2C-like state in ESCs.

By bioinformatically preselecting candidates based on their expression pattern and gene ontology, we were able to enrich for 2C-like regulators in our candidate-based screen. It will be exciting to follow up on the other screen hits to determine whether they may be part of a larger mechanism working either in parallel or together with Dppa2 and Dppa4 to regulate this crucial developmental progression. Interestingly, consistent with a previous report (Huang et al. 2017), the Dppa family member Dppa3 (also known as Stella or Pgc7) was not able to induce the 2C-like state despite activating MERVL elements in embryos (Huang et al. 2017). This may be explained by cofactors present in the oocyte but absent in ESCs and required for Dppa3 function and/or differences in chromatin structure.

Dppa2 and Dppa4 physically interact and bind to euchromatin in PSCs (Nakamura et al. 2011; Klein et al. 2018). Here, we show that Dppa2 and Dppa4 are regulated by DNA methylation and are necessary to induce an early embryonic transcriptional network by directly binding and regulating the ZGA transcription factor Dux in ESCs. Given the high perinatal lethality in the knockout and maternal stores of Dppa2 and Dppa4, it has not yet been possible to investigate whether Dppa2 and/or Dppa4 are required for ZGA in vivo. However, rare surviving Dppa4 knockout females have impaired fertility, yet germ cell development appears unaltered (Madan et al. 2009), suggesting potential important roles for this protein in preimplantation development. Furthermore, Dux knockdown in vivo results in impaired early embryonic development and defective ZGA (De Iaco et al. 2017). Moreover, injection of a dominant-negative form of Dppa2 lacking the SAP domain into zygotes induces two-cell arrest (Hu et al. 2010), suggesting that our results showing that Dppa2 and Dppa4 regulate the ZGA transcriptional network in ESCs may also apply to embryos.

In summary, in this study, we performed a candidate-based screen to identify epigenetic and chromatin regulators of the 2C-like state and ZGA transcriptional program. Among these were Dppa2 and Dppa4, which act together to bind and up-regulate the ZGA transcription factor Dux, among other non-ZGA targets. Depleting Dppa2 and Dppa4 levels reduces the 2C-like population and ZGA transcription, which can be restored by reintroducing either both Dppa2 and Dppa4 together or the downstream factor Dux. In conclusion, our findings reveal important insights into the molecular mechanisms regulating ZGA transcription.

## Materials and methods

### Gateway cloning

Sequence-verified cDNA sequences lacking stop codons were amplified from plasmids purchased from Thermo Fisher using primers containing AttB1 and AttB2 sequences and cloned into the pDONR221 vector. Gateway cloning was then used to transfer the cDNA sequences into an in-house-built pDEST vector containing a CAG promoter and an in-frame C-terminal eGFP-coding sequence and blasticidin resistance by IRES fusion. Expression plasmids were sequence-verified by Sanger sequencing prior to use and are available on request.

### Cell culture and flow cytometry

E14 mouse ESCs were grown under standard serum/LIF conditions (DMEM, 4,500 mg/L glucose, 4 mM L-glutamine, 110 mg/L sodium pyruvate, 15% fetal bovine serum, 1 U/mL penicillin, 1 mg/mL streptomycin, 0.1 mM nonessential amino acids, 50 mM b-mercaptoethanol, 10^3^ U/mL LIF). Single-MERVL::tdTomato and double-MERVL::tdTomato/Zscan4c::eGFP reporter cell lines were described in Eckersley-Maslin et al. (2016), and Dux knockout cells were described in De Iaco et al. (2017). Transfections were performed using Lipofectamine on preplated cells in six-well or 10-cm plate formats. Flow cytometry analysis was performed using BD LSR Fortessa, and sorts were performed on a BD Aria III or BD Influx high-speed cell sorter. siRNA transfections were performed by transfecting Dharmacon siRNA ON-TARGETplus siRNA SMARTpool at a final concentration of 50 nM with Lipofectamine.

### Generation of Dppa2 and Dppa4 CRISPR knockout ESCs

CRISPR knockout ESCs were performed as described previously (Ran et al. 2013). Guide RNAs were designed against exons 2 and 3 of both Dppa2 and Dppa4 using CRISPR design (http://crispr.mit.edu). Cells were transfected with a single guide targeting Dppa2 and/or Dppa4 and FACS (fluorescence-activated cell sorting)-sorted after 24 h into single cells, and clones were screened by surveyor assay and genomic DNA PCR. Successfully targeted clones were validated by Western blotting. Dppa2 single-knockout clones (clone 5 and clone 12) used in this study were generated using a guide RNA targeting Dppa2 exon 2 (ACCTTAGACCACACACCACCAGG), Dppa4 single-knockout clones (clone 23 and clone 29) were generated with a guide RNA targeting Dppa4 exon 2 (CTGCAAAGGCTAAAGCAACGGGG), and Dppa2/Dppa4 double-knockout clone (clone 43) was generated using a guide targeting Dppa2 exon 3 (TAACTTGAGTACGGATGGCAAGG) together with the guide RNA targeting Dppa4 (as above).

### RNA isolation, qPCR, and RNA-seq

RNA was isolated using Qiagen RNA–DNA allprep columns or TriReagent (Sigma) and treated with DNase I (Ambion DNA-free DNA [1311027] or Thermo Fisher RNase-free [EN0525]) following the manufacturer's instructions. cDNA was generated using 0.5–1 µg of RNA (Thermo RevertAid, K1622), and qRT–PCR was performed using Brilliant III SYBR master mix (Agilent Technologies, 600882). Relative quantification was performed using the comparative CT method with normalization to CycloB1 levels. Primer sequences are available on request. Opposite strand-specific polyA RNA libraries were made using 1 µg of DNase-treated RNA at the Sanger Institute Illumina bespoke pipeline and sequenced as single-end 50-base-pair (bp) reads using the Illumina HiSeq 2500 Rapid Run platform.

### Candidate screen selection

Candidate 2C-like ZGA regulators were selected based on the following criteria. First, genes expressed in oocytes/zygotes (reads per kilobase per million [RPKM] > 1 in Deng et al. 2014 data) were selected and further filtered based on gene ontology (AmiGO Ontology search for “chromatin”-associated genes). This gave a total list of 84 candidate genes that was manually curated to remove nonnuclear proteins and overlapping transcripts. Genes were finally filtered to include those that had sequence-verified cDNA clones commercially available.

### Data analysis

Raw FastQ data were trimmed with Trim Galore (version 0.4.3, default parameters) and mapped to the mouse GRCm38 genome assembly using Hisat2 version 2.0.5. Data were quantitated at the mRNA level using the RNA-seq quantitation pipeline in SeqMonk software (http://www.bioinformatics.babraham.ac.uk/projects/seqmonk). Strand-specific quantification was performed using mRNA probes and cumulative distributions matched across samples. Differentially expressed genes were determined using DESeq2 (*P*-value of 0.05 with multiple testing correction) and intensity difference filter (*P*-value of 0.05), with the high-confidence differentially expressed genes defined as the intersection between the two statistical tests. For Dppa2/Dppa4 single- and double-knockout transcriptome analysis, as there was only one double-knockout ESC clone, differentially expressed genes were defined independently in Dppa2-knockout and Dppa4 knockout ESCs, and the two lists were combined to get the “Dppa2^−/−^ or Dppa4^−/−^” differentially expressed genes.

### DNA methylation and ChIP-seq analysis

Published data sets were analyzed using SeqMonk software. For DNA methylation wiggle plots, the percentage methylation of individual CpGs with at least four reads are shown. ChIP-seq wiggle plots show normalized read counts for 10-bp running windows. For quantification of at gene promoters, probes were generated over TSSs ±500 bp, and the percentage DNA methylation or normalized ChIP-seq read count of the entire probe was calculated.

### Analysis of Dux

The mouse *dux* ORF is part of a 5-kb repeat unit that is organized as a large tandem array, with repeat numbers being polymorphic between strains and within outbred mice (Clapp et al. 2007). In the current genome assembly (GRCm38.p6), the protein-coding *duxf3* is located on a patch (CHR_MG4264_PATCH: 58,251, 173–58,259,474) with several homologs and pseudogenes present (Leidenroth and Hewitt 2010). To minimize confound by genomic multimapping in the analysis of this gene, we mapped RNA-seq, bisulfite sequencing (BS-seq), and ChIP-seq data directly against the mouse *dux* repeat (AM398147.1). Read counts were normalized to the total read count of the sample.

### Analysis of repeats

For alignments to repetitive regions in the genome, we constructed artificial repeat genomes. Repeat annotations were downloaded from the University of California at Santa Cruz (UCSC) browser (RepeatMasker, mm10, November 2018) and filtered for long instances of MERVL, MaLR, IAP, and LINE1 elements as well as major satellites (see Table 1). The length cutoff was introduced to enrich for functional full-length elements and exclude fragmented/truncated instances. Sequences of the filtered list of repeat element instances were stitched together, separated by “NNNNN” to create repeat-specific genomes. Trimmed reads from each sample were aligned against all individual repeat genomes using Bowtie2 (version 2.3.2). Values given are cumulated reads mapping to a specific repeat group as the percentage of the total read count.

**Table 1.**
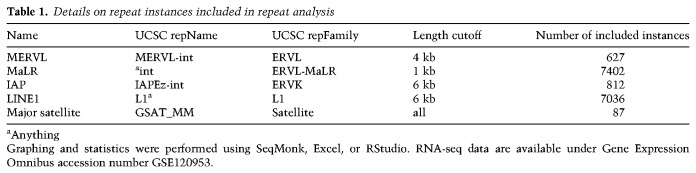
Details on repeat instances included in repeat analysis

### Western blotting

Western blotting was performed using 50 µg of protein extracted using detergent buffer (10 mM Tris-HCl at pH 7.4, 150 mM NaCl, 10 mM KCl, 0.5% NP-40) containing a protease inhibitor cocktail (Sigma, P2714) and quantified by Bio-Rad protein assay dye reagent. Proteins were resolved using 4%–12% SDS-PAGE gels (Expedon, NBT41212) and blotted on PVDF membranes. Following blocking in 5% skim milk/0.01% Tween/PBS, membranes were incubated with primary antibodies for 3 h to overnight. Secondary horseradish peroxidase-conjugated secondary antibodies (1:3000; Santa Cruz Biotechnology) were incubated for 1 h, and detection was carried out with enhanced chemiluminescence (ECL) reaction (GE Healthcare, RPN2209). The primary antibodies used were anti-Zscan4 (1:500; Millipore, 2793611), anti-Dppa2 (1:500; Millipore MAB4356), Dppa4 (1:200; Santa Cruz Biotechnology, sc-74614), and anti-HSP90 (1:5000; Abcam, ab13492).

## Supplementary Material

Supplemental Material

## References

[GAD321174ECKC1] Akiyama T, Xin L, Oda M, Sharov AA, Amano M, Piao Y, Cadet JS, Dudekula DB, Qian Y, Wang W, 2015 Transient bursts of Zscan4 expression are accompanied by the rapid derepression of heterochromatin in mouse embryonic stem cells. DNA Res 22: 307–318. 10.1093/dnares/dsv013 26324425 PMC4596397

[GAD321174ECKC2] Amano T, Hirata T, Falco G, Monti M, Sharova LV, Amano M, Sheer S, Hoang HG, Piao Y, Stagg CA, 2013 Zscan4 restores the developmental potency of embryonic stem cells. Nat Commun 4: 1966 10.1038/ncomms2966 23739662 PMC3682791

[GAD321174ECKC3] Auclair G, Guibert S, Bender A, Weber M. 2014 Ontogeny of CpG island methylation and specificity of DNMT3 methyltransferases during embryonic development in the mouse. Genome Biol 15: 545 10.1186/s13059-014-0545-5 25476147 PMC4295324

[GAD321174ECKC4] Bortvin A, Eggan K, Skaletsky H, Akutsu H, Berry DL, Yanagimachi R, Page DC, Jaenisch R. 2003 Incomplete reactivation of Oct4-related genes in mouse embryos cloned from somatic nuclei. Development 130: 1673–1680. 10.1242/dev.00366 12620990

[GAD321174ECKC5] Bošković A, Eid A, Pontabry J, Ishiuchi T, Spiegelhalter C, Raghu Ram EVS, Meshorer E, Torres-Padilla M-E. 2014 Higher chromatin mobility supports totipotency and precedes pluripotency in vivo. Genes Dev 28: 1042–1047. 10.1101/gad.238881.114 24831699 PMC4035533

[GAD321174ECKC6] Campbell AE, Shadle SC, Jagannathan S, Lim J-W, Resnick R, Tawil R, van der Maarel SM, Tapscott SJ. 2018 NuRD and CAF-1-mediated silencing of the D4Z4 array is modulated by DUX4-induced MBD3L proteins. Elife 7: e310233 10.7554/eLife.31023 PMC5849414 29533181

[GAD321174ECKC7] Choi YJ, Lin C-P, Risso D, Chen S, Kim TA, Tan MH, Li JB, Wu Y, Chen C, Xuan Z, 2017 Deficiency of microRNA miR-34a expands cell fate potential in pluripotent stem cells. Science 355: eaag1927 10.1126/science.aag1927 28082412 PMC6138252

[GAD321174ECKC8] Clapp J, Mitchell LM, Bolland DJ, Fantes J, Corcoran AE, Scotting PJ, Armour JAL, Hewitt JE. 2007 Evolutionary conservation of a coding function for D4Z4, the tandem DNA repeat mutated in facioscapulohumeral muscular dystrophy. Am J Hum Genet 81: 264–279. 10.1086/519311 17668377 PMC1950813

[GAD321174ECKC9] Dan J, Liu Y, Liu N, Chiourea M, Okuka M, Wu T, Ye X, Mou C, Wang L, Wang L, 2014 Rif1 maintains telomere length homeostasis of ESCs by mediating heterochromatin silencing. Dev Cell 29: 7–19. 10.1016/j.devcel.2014.03.004 24735877 PMC4720134

[GAD321174ECKC10] Dan J, Yang J, Liu Y, Xiao A, Liu L. 2015 Roles for histone acetylation in regulation of telomere elongation and two-cell state in mouse ES cells. J Cell Physiol 230: 2337–2344. 10.1002/jcp.24980 25752831 PMC4711819

[GAD321174ECKC11] Dan J, Rousseau P, Hardikar S, Veland N, Wong J, Autexier C, Chen T. 2017 Zscan4 inhibits maintenance DNA methylation to facilitate telomere elongation in mouse embryonic stem cells. Cell Rep 20: 1936–1949. 10.1016/j.celrep.2017.07.070 28834755 PMC5595351

[GAD321174ECKC12] De Iaco A, Planet E, Coluccio A, Verp S, Duc J, Trono D. 2017 DUX-family transcription factors regulate zygotic genome activation in placental mammals. Nat Genet 49: 941–945. 10.1038/ng.3858 28459456 PMC5446900

[GAD321174ECKC13] Deng Q, Ramsköld D, Reinius B, Sandberg R. 2014 Single-cell RNA-seq reveals dynamic, random monoallelic gene expression in mammalian cells. Science 343: 193–196. 10.1126/science.1245316 24408435

[GAD321174ECKC14] Eckersley-Maslin MA, Svensson V, Krueger C, Stubbs TM, Giehr P, Krueger F, Miragaia RJ, Kyriakopoulos C, Berrens RV, Milagre I, 2016 MERVL/Zscan4 network activation results in transient genome-wide DNA demethylation of mESCs. Cell Rep 17: 179–192. 10.1016/j.celrep.2016.08.087 27681430 PMC5055476

[GAD321174ECKC15] Eckersley-Maslin MA, Alda-Catalinas C, Reik W. 2018 Dynamics of the epigenetic landscape during the maternal-to-zygotic transition. Nat Rev Mol Cell Biol 19: 436–450. 10.1038/s41580-018-0008-z 29686419

[GAD321174ECKC16] Engelen E, Brandsma JH, Moen MJ, Signorile L, Dekkers DHW, Demmers J, Kockx CEM, Ozgür Z, van IJcken WFJ, van den Berg DLC, 2015 Proteins that bind regulatory regions identified by histone modification chromatin immunoprecipitations and mass spectrometry. Nat Commun 6: 7155 10.1038/ncomms8155 25990348 PMC4455091

[GAD321174ECKC17] Fujii S, Nishikawa-Torikai S, Futatsugi Y, Toyooka Y, Yamane M, Ohtsuka S, Niwa H. 2015 Nr0b1 is a negative regulator of Zscan4c in mouse embryonic stem cells. Sci Rep 5: 9146 10.1038/srep09146 25772165 PMC5390923

[GAD321174ECKC18] Hendrickson PG, Doráis JA, Grow EJ, Whiddon JL, Lim J-W, Wike CL, Weaver BD, Pflueger C, Emery BR, Wilcox AL, 2017 Conserved roles of mouse DUX and human DUX4 in activating cleavage-stage genes and MERVL/HERVL retrotransposons. Nat Genet 49: 925–934. 10.1038/ng.3844 28459457 PMC5703070

[GAD321174ECKC19] Hernandez C, Wang Z, Ramazanov B, Tang Y, Mehta S, Dambrot C, Lee Y-W, Tessema K, Kumar I, Astudillo M, 2018 Dppa2/4 facilitate epigenetic remodeling during reprogramming to pluripotency. Cell Stem Cell 23: 396–411.e8. 10.1016/j.stem.2018.08.001 30146411 PMC6128737

[GAD321174ECKC20] Hirata T, Amano T, Nakatake Y, Amano M, Piao Y, Hoang HG, Ko MSH. 2012 Zscan4 transiently reactivates early embryonic genes during the generation of induced pluripotent stem cells. Sci Rep 2: 208 10.1038/srep00208 22355722 PMC3250575

[GAD321174ECKC21] Hisada K, Sánchez C, Endo TA, Endoh M, Román-Trufero M, Sharif J, Koseki H, Vidal M. 2012 RYBP represses endogenous retroviruses and preimplantation- and germ line-specific genes in mouse embryonic stem cells. Mol Cell Biol 32: 1139–1149. 10.1128/MCB.06441-11 22269950 PMC3295013

[GAD321174ECKC22] Hu J, Wang F, Zhu X, Yuan Y, Ding M, Gao S. 2010 Mouse ZAR1-like (XM_359149) colocalizes with mRNA processing components and its dominant-negative mutant caused two-cell-stage embryonic arrest. Dev Dyn 239: 407–424. 10.1002/dvdy.22170 20014101

[GAD321174ECKC23] Huang Y, Kim JK, Do DV, Lee C, Penfold CA, Zylicz JJ, Marioni JC, Hackett JA, Surani MA. 2017 STELLA modulates transcriptional and endogenous retrovirus programs during maternal-to-zygotic transition. Elife 6: e22345 10.7554/eLife.22345 28323615 PMC5404928

[GAD321174ECKC24] Ishiuchi T, Torres-Padilla M-E. 2013 Towards an understanding of the regulatory mechanisms of totipotency. Curr Opin Genet Dev 23: 512–518. 10.1016/j.gde.2013.06.006 23942314

[GAD321174ECKC25] Ishiuchi T, Enriquez-Gasca R, Mizutani E, Bošković A, Ziegler-Birling C, Rodriguez-Terrones D, Wakayama T, Vaquerizas JM, Torres-Padilla M-E. 2015 Early embryonic-like cells are induced by downregulating replication-dependent chromatin assembly. Nat Struct Mol Biol 22: 662–671. 10.1038/nsmb.3066 26237512

[GAD321174ECKC26] Jukam D, Shariati SAM, Skotheim JM. 2017 Zygotic genome activation in vertebrates. Dev Cell 42: 316–332. 10.1016/j.devcel.2017.07.026 28829942 PMC5714289

[GAD321174ECKC27] Klein RH, Tung P-Y, Somanath P, Fehling HJ, Knoepfler PS. 2018 Genomic functions of developmental pluripotency associated factor 4 (Dppa4) in pluripotent stem cells and cancer. Stem Cell Res 31: 83–94. 10.1016/j.scr.2018.07.009 30031967 PMC6133722

[GAD321174ECKC28] Lee HJ, Hore TA, Reik W. 2014 Reprogramming the methylome: erasing memory and creating diversity. Cell Stem Cell 14: 710–719. 10.1016/j.stem.2014.05.008 24905162 PMC4051243

[GAD321174ECKC29] Leidenroth A, Hewitt JE. 2010 A family history of DUX4: phylogenetic analysis of DUXA, B, C and Duxbl reveals the ancestral DUX gene. BMC Evol Biol 10: 364 10.1186/1471-2148-10-364 21110847 PMC3004920

[GAD321174ECKC30] Li L, Lu X, Dean J. 2013 The maternal to zygotic transition in mammals. Mol Aspects Med 34: 919–938. 10.1016/j.mam.2013.01.003 23352575 PMC3669654

[GAD321174ECKC31] Macfarlan TS, Gifford WD, Agarwal S, Driscoll S, Lettieri K, Wang J, Andrews SE, Franco L, Rosenfeld MG, Ren B, 2011 Endogenous retroviruses and neighboring genes are coordinately repressed by LSD1/KDM1A. Genes Dev 25: 594–607. 10.1101/gad.2008511 21357675 PMC3059833

[GAD321174ECKC32] Macfarlan TS, Gifford WD, Driscoll S, Lettieri K, Rowe HM, Bonanomi D, Firth A, Singer O, Trono D, Pfaff SL. 2012 Embryonic stem cell potency fluctuates with endogenous retrovirus activity. Nature 487: 57–63. 10.1038/nature11244 22722858 PMC3395470

[GAD321174ECKC33] Madan B, Madan V, Weber O, Tropel P, Blum C, Kieffer E, Viville S, Fehling HJ. 2009 The pluripotency-associated gene Dppa4 is dispensable for embryonic stem cell identity and germ cell development but essential for embryogenesis. Mol Cell Biol 29: 3186–3203. 10.1128/MCB.01970-08 19332562 PMC2682008

[GAD321174ECKC34] Maenohara S, Unoki M, Toh H, Ohishi H, Sharif J, Koseki H, Sasaki H. 2017 Role of UHRF1 in de novo DNA methylation in oocytes and maintenance methylation in preimplantation embryos. PLoS Genet 13: e1007042 10.1371/journal.pgen.1007042 28976982 PMC5643148

[GAD321174ECKC35] Maksakova IA, Thompson PJ, Goyal P, Jones SJ, Singh PB, Karimi MM, Lorincz MC. 2013 Distinct roles of KAP1, HP1 and G9a/GLP in silencing of the two-cell-specific retrotransposon MERVL in mouse ES cells. Epigenetics Chromatin 6: 15 10.1186/1756-8935-6-15 23735015 PMC3682905

[GAD321174ECKC36] Maldonado-Saldivia J, van den Bergen J, Krouskos M, Gilchrist M, Lee C, Li R, Sinclair AH, Surani MA, Western PS. 2007 Dppa2 and Dppa4 are closely linked SAP motif genes restricted to pluripotent cells and the germ line. Stem Cells 25: 19–28. 10.1634/stemcells.2006-0269 16990585

[GAD321174ECKC37] Masaki H, Nishida T, Kitajima S, Asahina K, Teraoka H. 2007 Developmental pluripotency-associated 4 (DPPA4) localized in active chromatin inhibits mouse embryonic stem cell differentiation into a primitive ectoderm lineage. J Biol Chem 282: 33034–33042. 10.1074/jbc.M703245200 17855347

[GAD321174ECKC38] Masaki H, Nishida T, Sakasai R, Teraoka H. 2010 DPPA4 modulates chromatin structure via association with DNA and core histone H3 in mouse embryonic stem cells. Genes Cells 15: 327–337. 10.1111/j.1365-2443.2010.01382.x 20298437

[GAD321174ECKC39] Milagre I, Stubbs TM, King MR, Spindel J, Santos F, Krueger F, Bachman M, Segonds-Pichon A, Balasubramanian S, Andrews SR, 2017 Gender differences in global but not targeted demethylation in iPSC reprogramming. Cell Rep 18: 1079–1089. 10.1016/j.celrep.2017.01.008 28147265 PMC5300890

[GAD321174ECKC40] Mohammed H, Hernando-Herraez I, Savino A, Scialdone A, Macaulay I, Mulas C, Chandra T, Voet T, Dean W, Nichols J, 2017 Single-cell landscape of transcriptional heterogeneity and cell fate decisions during mouse early gastrulation. Cell Rep 20: 1215–1228. 10.1016/j.celrep.2017.07.009 28768204 PMC5554778

[GAD321174ECKC41] Nakamura T, Nakagawa M, Ichisaka T, Shiota A, Yamanaka S. 2011 Essential roles of ECAT15-2/Dppa2 in functional lung development. Mol Cell Biol 31: 4366–4378. 10.1128/MCB.05701-11 21896782 PMC3209334

[GAD321174ECKC42] Nishiyama A, Xin L, Sharov AA, Thomas M, Mowrer G, Meyers E, Piao Y, Mehta S, Yee S, Nakatake Y, 2009 Uncovering early response of gene regulatory networks in ESCs by systematic induction of transcription factors. Cell Stem Cell 5: 420–433. 10.1016/j.stem.2009.07.012 19796622 PMC2770715

[GAD321174ECKC43] Percharde M, Lin C-J, Yin Y, Guan J, Peixoto GA, Bulut-Karslioglu A, Biechele S, Huang B, Shen X, Ramalho-Santos M. 2018 A LINE1-nucleolin partnership regulates early development and ESC identity. Cell 174: 391–405.e19. 10.1016/j.cell.2018.05.043 29937225 PMC6046266

[GAD321174ECKC44] Pfeiffer MJ, Siatkowski M, Paudel Y, Balbach ST, Baeumer N, Crosetto N, Drexler HCA, Fuellen G, Boiani M. 2011 Proteomic analysis of mouse oocytes reveals 28 candidate factors of the ‘reprogrammome’. J Proteome Res 10: 2140–2153. 10.1021/pr100706k 21344949

[GAD321174ECKC45] Ran FA, Hsu PD, Wright J, Agarwala V, Scott DA, Zhang F. 2013 Genome engineering using the CRISPR–Cas9 system. Nat Protoc 8: 2281–2308. 10.1038/nprot.2013.143 24157548 PMC3969860

[GAD321174ECKC46] Rodriguez-Terrones D, Gaume X, Ishiuchi T, Weiss AXL, Kopp A, Kruse K, Penning A, Vaquerizas JM, Brino L, Torres-Padilla M-E. 2017 A molecular roadmap for the emergence of early-embryonic-like cells in culture. Nat Genet 50: 106–119. 10.1038/s41588-017-0016-5 29255263 PMC5755687

[GAD321174ECKC47] Rowe HM, Jakobsson J, Mesnard D, Rougemont J, Reynard S, Aktas T, Maillard PV, Layard-Liesching H, Verp S, Marquis J, 2010 KAP1 controls endogenous retroviruses in embryonic stem cells. Nature 463: 237–240. 10.1038/nature08674 20075919

[GAD321174ECKC48] Schoorlemmer J, Pérez-Palacios R, Climent M, Guallar D, Muniesa P. 2014 Regulation of mouse retroelement MuERV-L/MERVL expression by REX1 and epigenetic control of stem cell potency. Front Oncol 4: 14 10.3389/fonc.2014.00014 24567914 PMC3915180

[GAD321174ECKC49] Storm MP, Kumpfmueller B, Bone HK, Buchholz M, Sanchez Ripoll Y, Chaudhuri JB, Niwa H, Tosh D, Welham MJ. 2014 Zscan4 is regulated by PI3-kinase and DNA-damaging agents and directly interacts with the transcriptional repressors LSD1 and CtBP2 in mouse embryonic stem cells. PLoS One 9: e89821 10.1371/journal.pone.0089821 24594919 PMC3940611

[GAD321174ECKC50] Svoboda P. 2018 Mammalian zygotic genome activation. Semin Cell Dev Biol 84: 118–126. 10.1016/j.semcdb.2017.12.006 29233752

[GAD321174ECKC51] Veselovska L, Smallwood SA, Saadeh H, Stewart KR, Krueger F, Maupetit-Méhouas S, Arnaud P, Tomizawa S-I, Andrews S, Kelsey G. 2015 Deep sequencing and de novo assembly of the mouse oocyte transcriptome define the contribution of transcription to the DNA methylation landscape. Genome Biol 16: 209 10.1186/s13059-015-0769-z 26408185 PMC4582738

[GAD321174ECKC52] Wang L, Zhang J, Duan J, Gao X, Zhu W, Lu X, Yang L, Zhang J, Li G, Ci W, 2014 Programming and inheritance of parental DNA methylomes in mammals. Cell 157: 979–991. 10.1016/j.cell.2014.04.017 24813617 PMC4096154

[GAD321174ECKC53] Whiddon JL, Langford AT, Wong C-J, Zhong JW, Tapscott SJ. 2017 Conservation and innovation in the DUX4-family gene network. Nat Genet 49: 935–940. 10.1038/ng.3846 28459454 PMC5446306

[GAD321174ECKC54] Xue Z, Huang K, Cai C, Cai L, Jiang C-Y, Feng Y, Liu Z, Zeng Q, Cheng L, Sun YE, 2013 Genetic programs in human and mouse early embryos revealed by single-cell RNA sequencing. Nature 500: 593–597. 10.1038/nature12364 23892778 PMC4950944

[GAD321174ECKC55] Zalzman M, Falco G, Sharova LV, Nishiyama A, Thomas M, Lee S-L, Stagg CA, Hoang HG, Yang H-T, Indig FE, 2010 Zscan4 regulates telomere elongation and genomic stability in ES cells. Nature 464: 858–863. 10.1038/nature08882 20336070 PMC2851843

[GAD321174ECKC56] Zhao T, Fu Y, Zhu J, Liu Y, Zhang Q, Yi Z, Chen S, Jiao Z, Xu X, Xu J, 2018 Single-cell RNA-Seq reveals dynamic early embryonic-like programs during chemical reprogramming. Cell Stem Cell 23: 31–45.e7. 10.1016/j.stem.2018.05.025 29937202

