# Supplementary material for: Dppa2 and Dppa4 directly regulate the Dux-driven zygotic transcriptional program
Source: Genes Dev. 2019 Feb 1;33(3-4):194–208. doi: 10.1101/gad.321174.118 (PMC6362816; doi:10.1101/gad.321174.118)
Supplement: Supplemental Material [file supp_33_3-4_194__index.html]

Dppa2 and Dppa4 directly regulate the Dux-driven zygotic transcriptional program — Supplemental Material 

# Dppa2 and Dppa4 directly regulate the Dux-driven zygotic transcriptional program

## Supplemental Material

- SupplementalTable1.xlsx
- SupplementalTable2.xlsx
- SupplementalTable3.xlsx
- SupplementalTable4.xlsx
- SupplementalTable5.xlsx
- SupplementalTable6.xlsx
- Supplemental\_Figure1.pdf
- Supplemental\_Figure2.pdf
- Supplemental\_Figure5.pdf
- Supplemental\_Figure3.pdf
- Supplemental\_Figure6.pdf
- Supplemental\_Figure4.pdf
- Supplemental\_Figure7.pdf
- Supplemental\_Figure\_Legends.docx
