# Supplementary material for: Dppa2 and Dppa4 directly regulate the Dux-driven zygotic transcriptional program
Source: Genes Dev. 2019 Feb 1;33(3-4):194–208. doi: 10.1101/gad.321174.118 (PMC6362816; doi:10.1101/gad.321174.118)

Supplemental Figure 1

A

	Zscan4	MERVL	Tcstv3	Gm8994	Zfp352	Gm5039	Gm428 cluster	Gm4014	gm4340/2123	Gm7647 cluster	tmem92	Eif1a/Eif1a-like
**Lhx2**	5.25	2.61	6.75	2.04	7.81	4.33	2.00	6.73	3.70	3.85	1.15	2.63
	13.50	2.95	13.44	8.20	7.24	9.88	11.90	8.42	9.47	11.56	2.84	8.66
**Npm2**	3.04	1.59	2.92	2.58	3.00	3.06	2.84	3.71	2.71	2.83	2.36	
	6.23	1.33	5.88	5.11	4.67	8.90	6.38	6.16	3.62	10.32	3.95	4.88
**Ss18l1**	6.20	1.89	5.31	4.94	6.03	3.85		10.79				
	2.39	1.34	0.80	0.83	0.66	0.62		0.80				2.63
	2.21	3.11	2.34	2.07	2.66	3.04	1.86	3.45	3.40	2.81	2.41	2.63
	10.98	2.62	5.97	4.00	0.95	2.03	2.28	7.32	4.31	4.36	1.02	6.13
**Dppa3**	2.38	2.09	2.85	2.99	2.99	3.34		2.71				1.91
	1.98	0.50	1.77	1.44	1.52	1.31	1.37	1.64	1.05	1.69	1.18	1.91
**Smad3**	2.36	2.11	1.90	2.74	2.22	2.79		3.97				
	5.43	2.43	3.89	5.01	2.46	7.26	5.37	2.45	2.85	3.47	3.35	6.23
	32.54	5.78	16.99	18.57	8.65	20.16	28.06	16.63	21.02	27.01	3.85	21.64
**Rb1**	7.25	1.98	3.93	4.98	3.15	7.17	7.02	3.47	3.71	6.42	3.55	7.72
	5.16	1.34	5.58	5.15	3.25	25.20	4.17	2.38	3.70	5.81	4.96	3.93
**Calcoco1**	4.87	2.11	3.05	4.59	3.16	2.74	2.77	2.36	2.43	2.41	1.99	7.72
	6.42	1.82	1.93	4.51	1.36	4.37	5.66	2.20	2.50	3.51	1.46	2.82
**Apbb1**	4.68	1.62	2.93	3.08	2.98	4.82	3.11	10.23	2.50	3.48	2.67	3.97
	4.06	2.34	5.22	3.31	4.85	3.51	4.00	2.54	14.99	2.84	2.51	5.90
**Plac8**	3.43	1.85	2.37	3.14	1.25	4.00	3.81	2.11	3.31	2.92	2.67	3.32
	6.13	2.83	7.26	4.84	9.80	8.87	5.81	9.70	18.97	5.94	3.28	7.54
**GFP**	2.65	1.66	3.67	2.35	2.37	2.65		1.85				
	2.64	1.44	2.82	2.71	2.99	2.92		2.22				3.50
	3.40	1.59	2.70	2.79	1.37	2.97	3.43	3.87	3.35	2.86	2.52	7.87
	3.23	1.58	1.81	3.51	1.30	1.98	4.57	3.70	3.79	3.22	1.65	5.22
	1.52	0.90	1.46	1.82	0.06	1.01	1.06	0.49	2.14	1.18	0.05	2.42
**Irf1**	6.89	2.58	4.98	2.31	14.42	2.11	2.84	7.62	2.29	5.96	19.16	21.06
	9.55	2.66	3.96	3.11	5.02	6.05	1.60	50.85	44.69	30.05	2.61	21.06
	11.07	2.05	2.08	1.39	14.70	8.24	1.03	16.13	1.47	8.61	41.59	
	17.96	3.93	7.85	6.51	10.70	14.17	6.83	19.05	6.64	17.08	13.39	23.15
**Mae1**	7.71	2.02	5.70	5.83	4.62	5.53	5.52	7.48	4.47	6.34	4.42	8.93
	2.62	1.45	5.68	4.85	1.81	3.99	2.48	1.12	1.74	2.73	1.05	2.10
**Eya1**	14.11	3.22	22.92	10.52	4.68	9.56	15.11	51.99	20.77	15.77	4.48	30.17
	13.47	3.19	12.78	9.97	6.11	14.87	9.19	7.26	8.84	8.52	8.27	3.23
	14.46	2.35	8.53	9.65	5.38	8.54	9.08	7.41	8.35	10.10	7.17	9.46
**Dppa2**	7.12	5.65	12.68	9.05	12.50	9.02	7.70	13.05	11.37	8.41	4.23	12.64
	7.04	3.62	5.18	5.73	8.44	5.52	6.33	4.22	6.67	5.75	4.17	6.20
	9.31	3.83	4.74	6.89	6.37	5.80	7.88	2.79	4.83	5.76	2.45	6.50
**Sp110**	6.62	1.91	6.16	6.56	6.43	11.59	6.08	1.58	1.95	5.16	4.36	7.14
	8.44	2.22	9.21	5.62	8.74	7.44	7.14	3.84	3.57	6.00	8.34	6.29
	3.52	3.11	3.17	3.25	4.22	3.94	4.07	5.10	5.25	5.75	3.06	5.00
	6.29	2.89	5.16	5.04	5.20	6.77	5.80	4.14	4.35	7.78	4.31	7.14
**Gata3**	4.03	2.33	11.43	5.88	15.62	2.39	1.11	6.66	3.23	3.53	29.24	5.94
	3.54	1.17	11.12	2.20	4.54	5.04	2.08	2.26	1.31	2.38	2.95	2.95
	0.86	0.76	1.70	0.92	1.34	0.67	0.63	0.64	0.64	0.60	0.72	0.91
	8.91	2.41	22.22	6.31	10.51	6.99	4.19	11.34	4.41	6.05	5.71	9.21
**Trp63**	86.10	4.49	16.70	9.72	10.75	40.44	13.88	4.91	2.65	48.54	13.91	54.07
	47.85	3.64	16.20	9.49	10.44	35.41	18.75	21.59	12.45	38.29	31.59	50.49
	11.99	1.47	2.49	1.60	2.19	8.79	10.92	4.99	2.43	24.46	4.47	15.04
	22.40	2.18	7.58	2.91	2.12	10.16	2.43	6.37	4.49	18.32	4.15	12.16
**Bahd1**	27.35	5.09	16.12	12.81	11.99	24.96	16.18	19.16	11.24	18.85	8.03	29.66
	7.53	3.55	6.17	6.31	6.93	13.06	12.76	12.45	9.13	11.74	3.68	11.93
	6.05	2.87	4.46	3.05	3.84	4.26	3.99	6.05	3.61	3.78	2.53	5.83
**Usp3**	12.04	7.81	9.38	10.70	7.31	11.20	11.08	11.27	11.75	11.67	6.92	15.03
	25.81	3.14	6.51	10.91	2.31	14.20	15.90	0.36	2.30	5.42	4.03	14.51
	30.37	8.23	25.66	20.04	15.73	19.93	22.64	19.31	25.45	13.61	8.06	21.91
**Tox3**	10.02	2.39	10.06	5.03	7.52	7.46	8.91	33.47	7.49	31.12	7.52	14.47
	16.02	3.82	17.12	5.34	10.69	9.63	7.55	34.78	9.29	54.19	11.89	2.63
	3.41	1.75	5.00	2.09	3.47	3.95	8.33	64.90	6.03	52.33	2.73	9.23
**Hdac9**	61.63	10.69	65.22	27.12	65.31	75.06	23.22	28.42	42.93	42.80	15.29	62.67
	10.68	2.37	7.33	6.37	4.43	11.20	4.02	3.99	9.89	7.84	2.27	18.81
	10.63	5.47	7.31	8.34	8.69	14.75	3.25	13.55	6.97	9.45	3.77	11.55
	8.40	1.94	5.66	3.99	1.68	8.17	1.68	1.21	1.30	4.65	0.80	6.12
**Dppa4**	22.59	12.55	30.00	20.29	43.60	20.21	10.50	17.26	14.26	22.82	13.51	27.31
	32.89	15.32	25.79	16.02	39.69	25.46	12.41	15.48	37.97	27.13	9.32	30.84
	27.46	11.16	24.63	24.30	25.81	21.35	11.31	12.71	30.37	22.73	9.27	27.82
**Zscan4c**	46.40	10.41	20.71	22.59	11.21	25.74	15.35	17.63	12.37	16.71	12.97	23.21
	53.37	11.63	73.43	56.12	29.04	42.06	34.96	29.32	25.57	36.85	26.74	43.13
	23.43	6.59	14.68	14.71	15.30	13.24	17.89	7.58	14.93	16.32	12.47	16.50

B

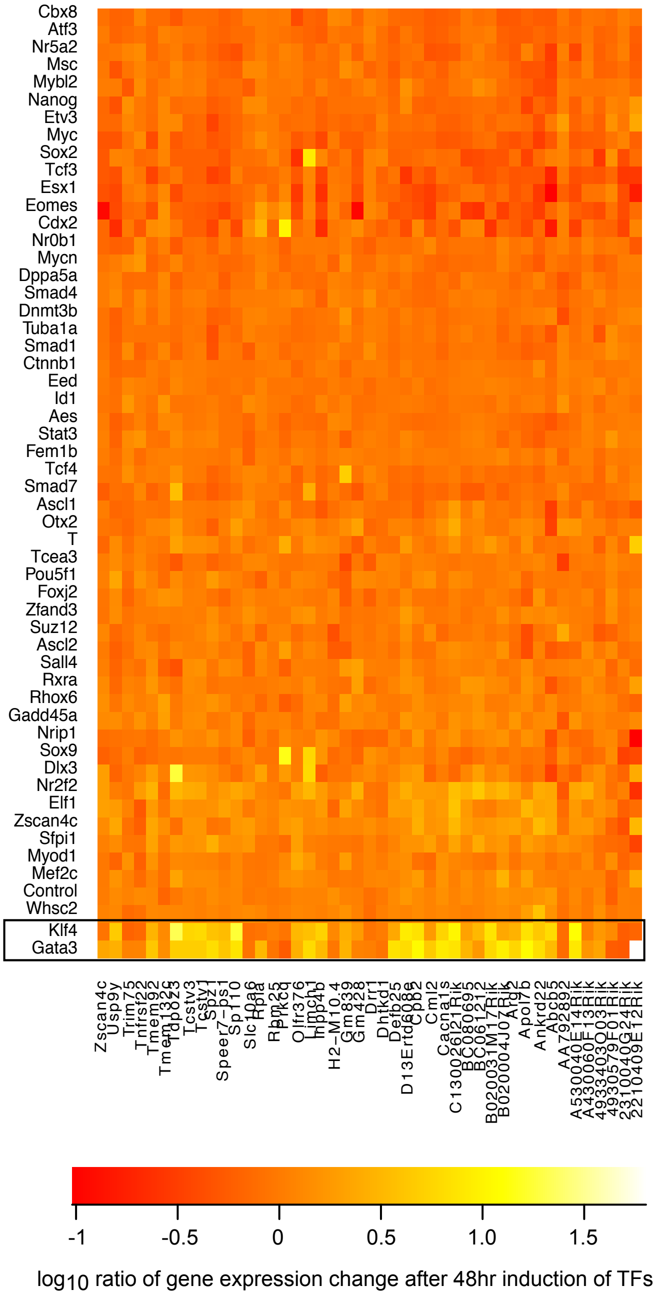

Supplement: Supplemental Material [file supp_gad.321174.118_Supplemental_Figure1.pdf]
