# Supplementary figures and images for: Dppa2 and Dppa4 directly regulate the Dux-driven zygotic transcriptional program
Source: Genes Dev. 2019 Feb 1;33(3-4):194–208. doi: 10.1101/gad.321174.118 (PMC6362816; doi:10.1101/gad.321174.118)

Supplemental Figure 2

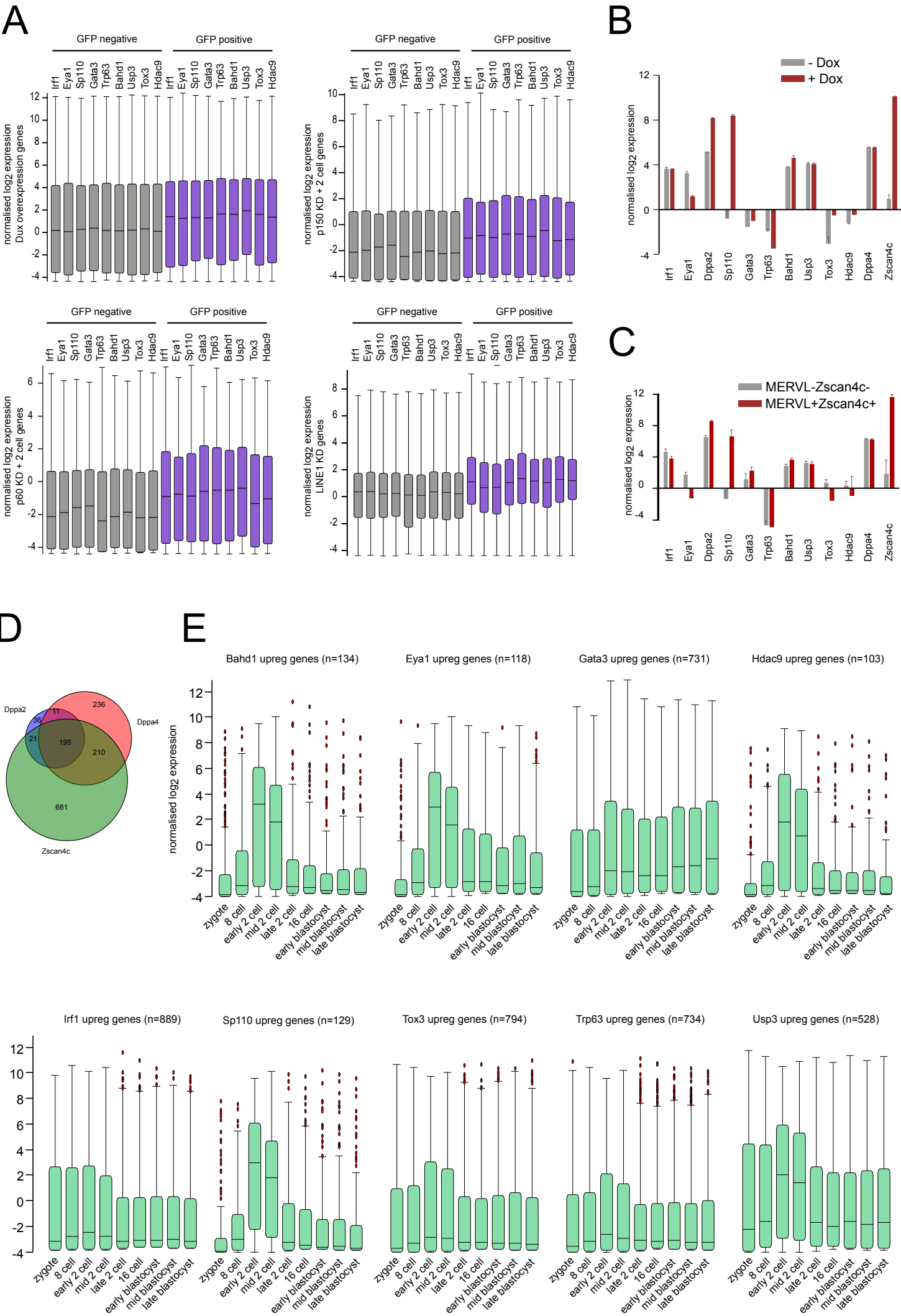

Supplement: Supplemental Material [file supp_gad.321174.118_Supplemental_Figure2.pdf]

# Supplemental Figure 5

## A

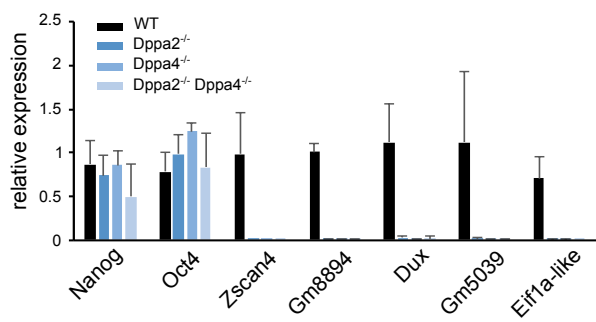

## B

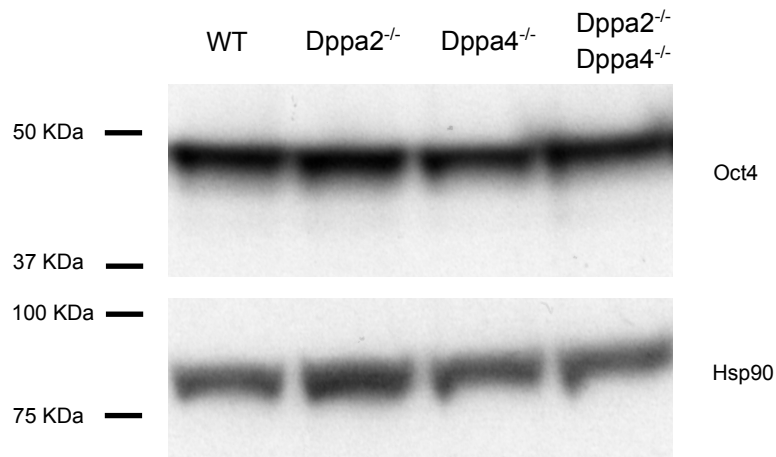

## C

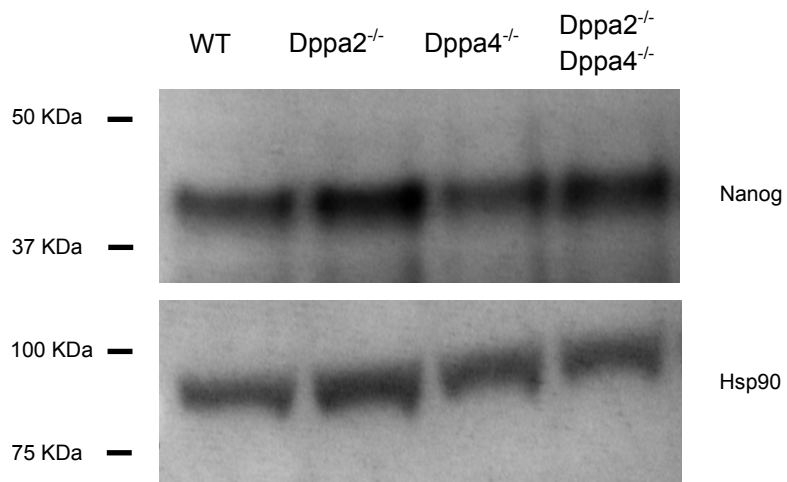

Supplement: Supplemental Material [file supp_gad.321174.118_Supplemental_Figure5.pdf]

# Supplemental Figure 3

A

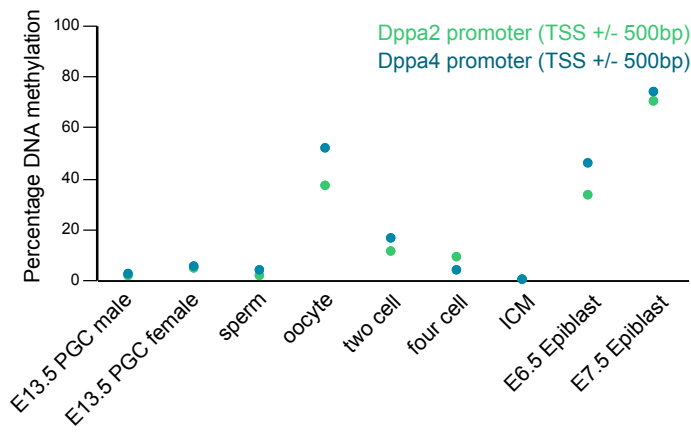

B

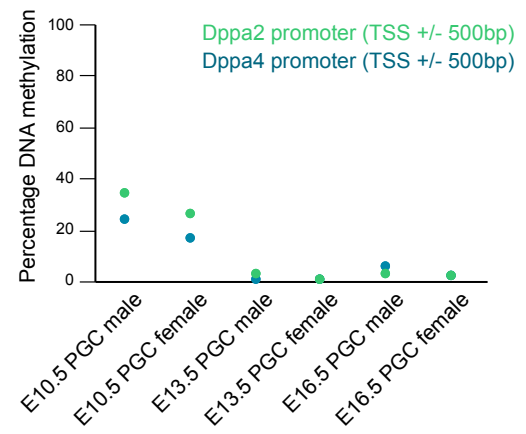

C

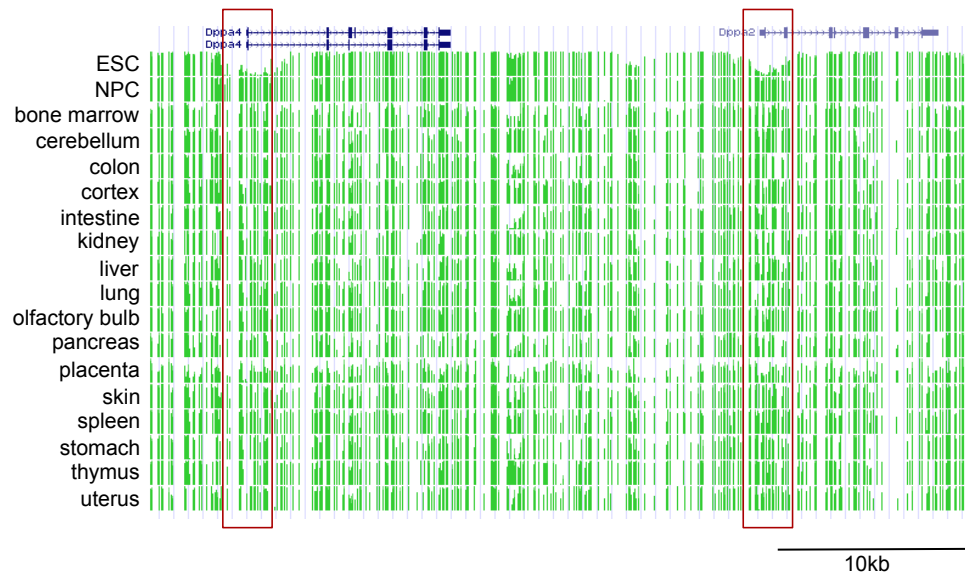

Supplement: Supplemental Material [file supp_gad.321174.118_Supplemental_Figure3.pdf]

# Supplemental Figure 6

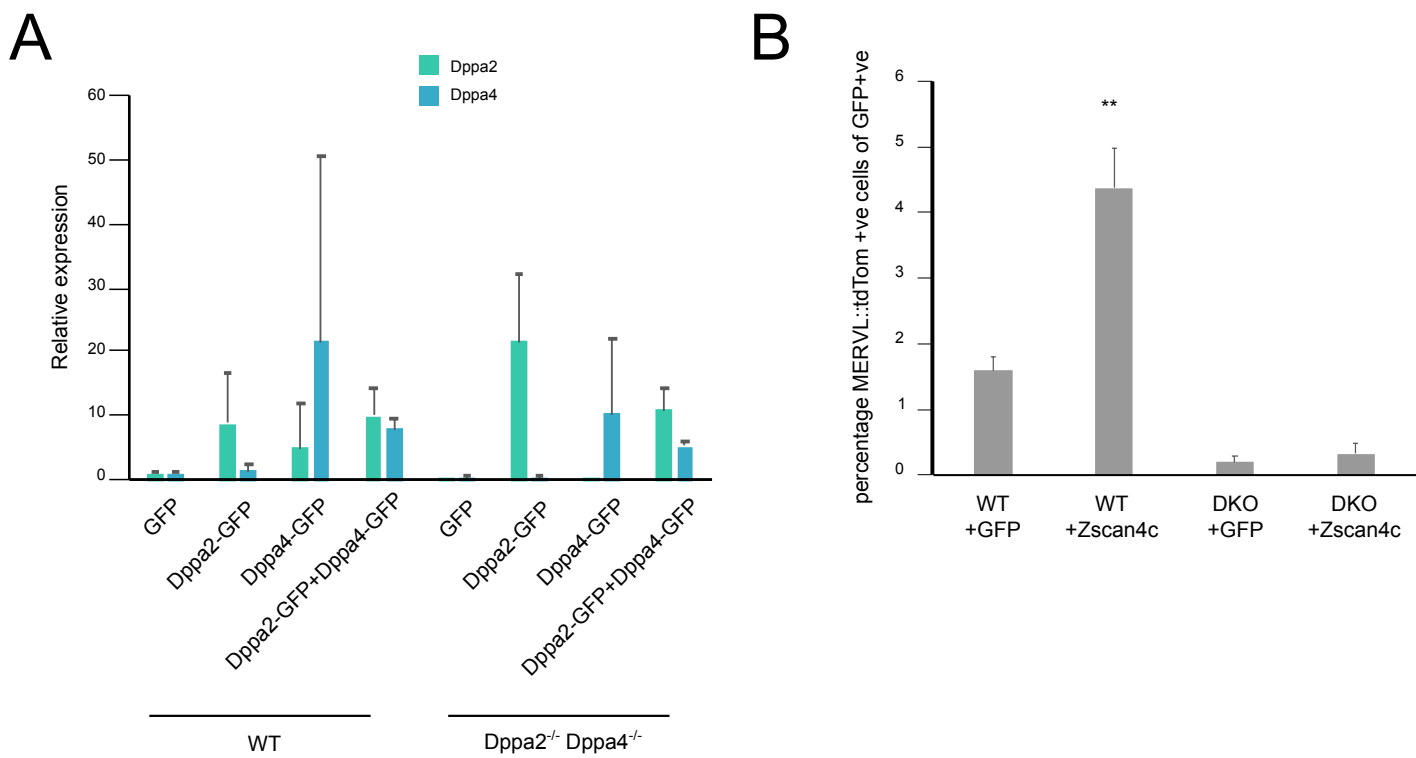

Supplement: Supplemental Material [file supp_gad.321174.118_Supplemental_Figure6.pdf]

# Supplemental Figure 4

A

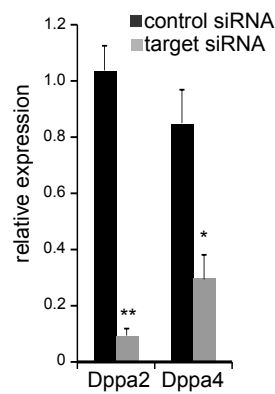

B

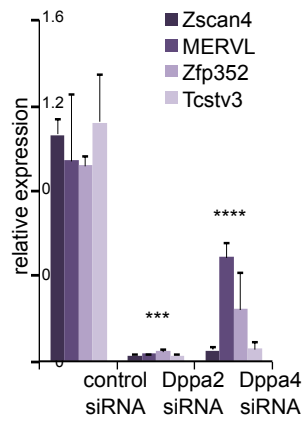

C

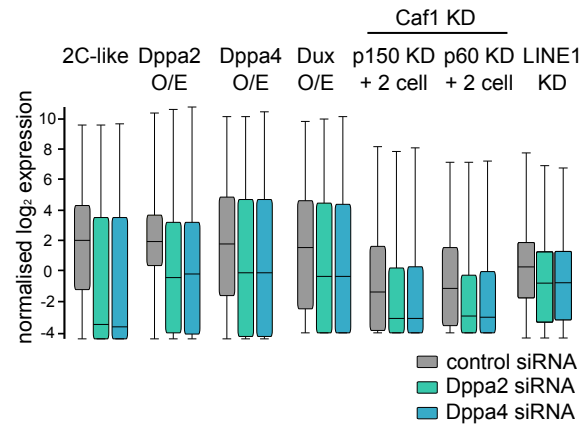

Supplement: Supplemental Material [file supp_gad.321174.118_Supplemental_Figure4.pdf]

Supplemental Figure 7

A

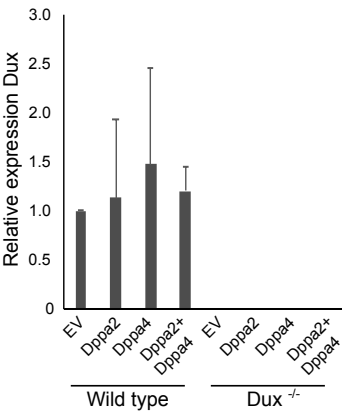

B

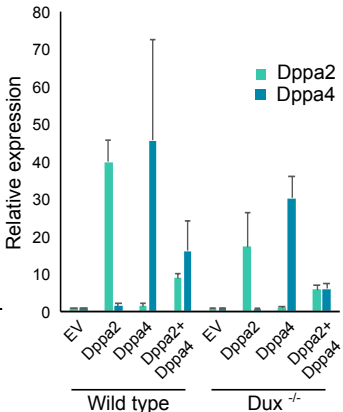

C

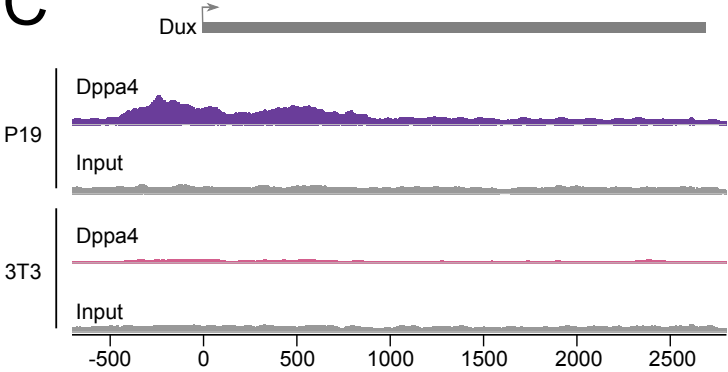

D

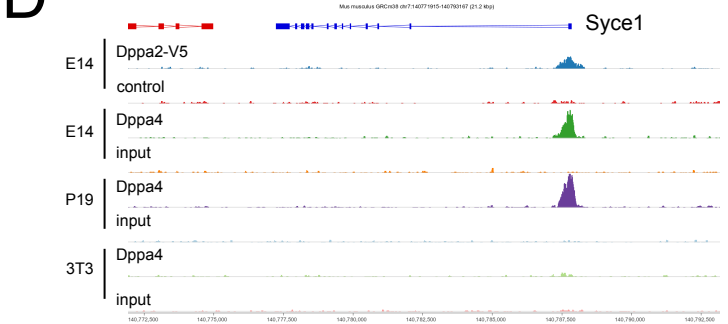

F

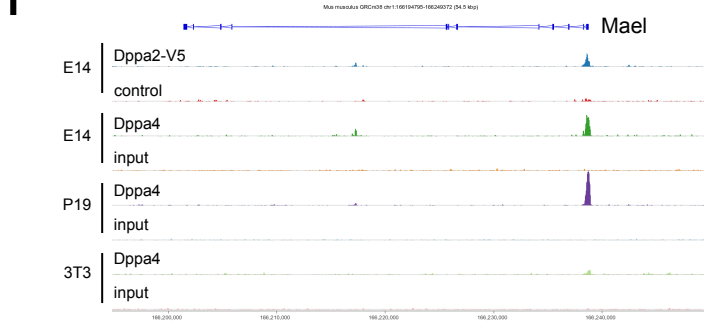

E

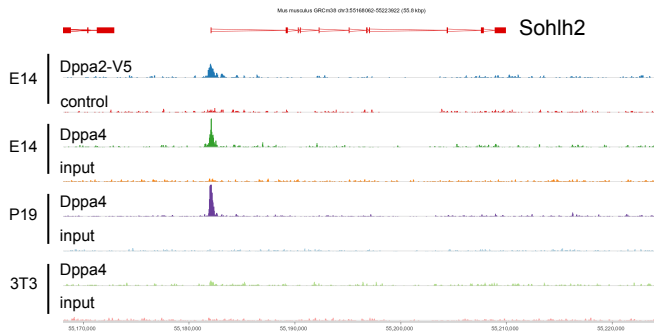

G

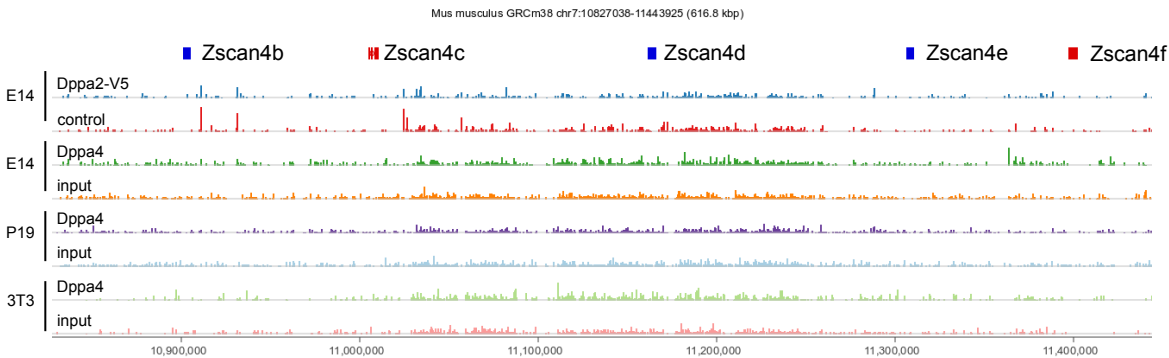

H

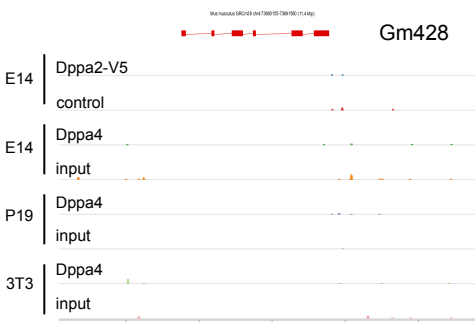

I

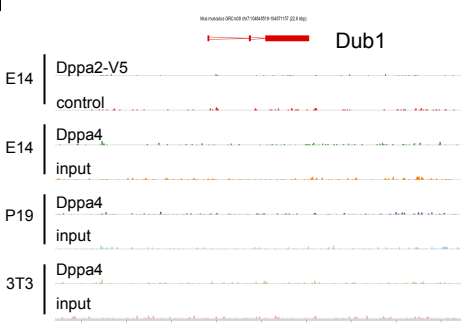

J

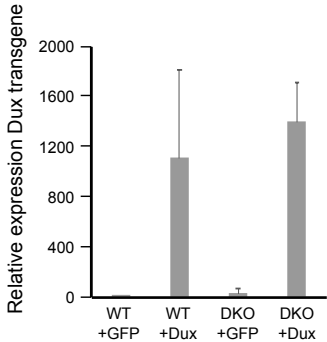

Supplement: Supplemental Material [file supp_gad.321174.118_Supplemental_Figure7.pdf]
