# Supplementary material for: Dppa2 and Dppa4 directly regulate the Dux-driven zygotic transcriptional program
Source: Genes Dev. 2019 Feb 1;33(3-4):194–208. doi: 10.1101/gad.321174.118 (PMC6362816; doi:10.1101/gad.321174.118)
Supplement: Supplemental Material [file supp_gad.321174.118_Supplemental_Figure_Legends.docx]

**SUPPLEMENTAL FIGURE LEGENDS**

**Supplemental Figure 1**

(A) Summary of quantitative RT-PCR results from screen. Fold changes of GFP-positive over GFP-negative sorted cells of a panel of ZGA transcript markers (columns) are shown and pseudocoloured from low (blue) to high (red). Screen candidates are in rows, each row represents an individual replicate. Blank cells = not assessed. GFP negative control is highlighted in green, screen hits in light blue, Dppa2 and Dppa4 in red and Zscan4c in dark blue (B) Log_10_ expression of ZGA transcripts (columns) by microarray analysis following 48 hours induction of various transcription factors (rows). Data reanalysed from (Nishiyama et al. 2009).

**Supplemental Figure 2**

(A) Box and Whisker plots showing expression of genes upregulated by Dux (top left, data reanalysed from (Hendrickson et al. 2017)), Caf1 p150 (top right) or p60 (bottom left) subunit knockdown (KD) (gene lists from (Ishiuchi et al. 2015)) or LINE1 knockdown (bottom right, gene list from (Percharde et al. 2018)) in GFP negative (grey) and GFP positive (purple) cells following transfection of GFP-tagged constructs of screen hits in ESCs. (B) Expression of candidates following Dux overexpression (+Dox, red) in ESCs compared to uninduced cells (-Dox, grey). Data reanalyzed from (Hendrickson et al. 2017). (C) Expression of candidates in 2C-like ESCs (MERVL+Zscan4c, red) compared to negative sorted cells (MERVL-Zscan4c-, grey). Data reanalyzed from (Eckersley-Maslin et al. 2016). (D) Wiggle plot showing HA-tagged Dux (top, blue) enrichment across the Dppa2/4 locus. Input (bottom, green) is shown as a control. Dppa2 and Dppa4 gene structures are shown in red. (E) Expression levels of IAP (left), MaLR (middle) and Major Satellite (right) repeat families following overexpression of screen candidates (black bars) compared to untransfected control cells (grey bars). Error bars represent average + standard deviation of at least 3 biological replicates. (F) Venn diagram showing overlap between differentially expressed genes following overexpression of Dppa2, Dppa4 and Zscan4c. (G) Expression pattern of genes upregulated following overexpression of screen hits during preimplantation development. Preimplantation data from (Deng et al. 2014).

**Supplemental Figure 3**

(A) Percentage DNA methylation at Dppa2 (green) and Dppa4 (blue) promoters (transcription start site (TSS) +/- 500bp) across embryonic tissues. Data reanalyzed from (Wang et al. 2014) except oocyte data which is reanalyzed from {Maenohara:2017jr}.. (B) Percentage DNA methylation at Dppa2 (green) and Dppa4 (blue) promoters (transcription start site (TSS) +/- 500bp) during PGC development *in vivo*. Data reanalyzed from (Kobayashi et al. 2012). (C) Box whisker plots showing percentage DNA methylation of all gene promoters (grey) compared to 2C-like gene promoters (red) during embryonic development. Promoters defined as TSS +/- 500bp. Data from {Wang:2014kf} except for oocyte data which is from {Maenohara:2017jr}. (D) DNA methylation landscape across Dppa2/Dppa4 locus in ESCs where they are expressed and a range of tissues where they are not expressed. Red box denotes approximate location of DMR. Data from DevMouse DNA methylome database (Liu et al. 2014)

**Supplemental Figure 4**

(A) Quantitative RT-PCR analysis showing knockdown efficiencies of Dppa2 (middle) and Dppa4 (right) siRNA compared to control siRNA (left). Error bars represent standard deviations of 3 biological replicates. Differences are statistically significant (homoscedastic two-tailed t-test, ** p-value <0.01, *** p-value < 0.001). (B) Quantitative RT-PCR analysis of ZGA transcripts in cells following treatment with control (left), Dppa2 (middle) or Dppa4 (right) siRNA. Pluripotency genes are shown in dark and light grey, Dux is highlighted in dark green. Error bars represent standard deviation of 3 biological replicates. Differences are statistically significant are denoted (* p-value < 0.05, ** p-value <0.01, *** p-value < 0.001, homoscedastic two-tailed t-test). (C) Expression levels of various genes lists in control (grey), Dppa2 (green) and Dppa4 (blue) siRNA treated cells. 2C-like transcripts defined by (Eckersley-Maslin et al. 2016), Dppa2 and Dppa4 overexpression (O/E) data defined in this manuscript, Dux overexpression (O/E) data reanalyzed from (Hendrickson et al. 2017), Caf1 p150 or p60 subunit knockdown (KD) transcripts also expressed in two cell embryo defined by (Ishiuchi et al. 2015) and LINE1 knockdown defined by (Percharde et al. 2018).

**Supplemental Figure 5**

(A) Expression levels of pluripotency (Nanog, Oct4) and ZGA (Zscan4, Gm8894, Dux, Gm5039, Eif1a-like) transcripts in WT (black), Dppa2^-/-^ (dark blue), Dppa4^-/-^ (medium blue) and Dppa2^-/-^Dppa4^-/-^ DKO (light blue) cells. Error bars represent standard deviations of at least 3 biological replicates. (B-C) Western blotting for (B) Oct4 and (C) Nanog in individual clones for wild type (WT), Dppa2^-/-^, Dppa4^-/-^ and double Dppa2^-/-^Dppa4^-/-^ (DKO) ESCs. Hsp90 is used as loading control. (D) Venn diagram showing overlap between Dppa2 KO and Dppa4 KO differentially expressed genes. (E) Venn diagram showing overlap between Dppa2 KO and Dppa2 siRNA differentially expressed genes (F) Venn diagram showing overlap between Dppa4 KO and Dppa4 siRNA differentially expressed genes. (G) Per probe normalized heatmap showing expression levels of all genes differentially expressed in either siRNA or CRISPR knockout experiments for either Dppa2 or Dppa4 across all samples. (H-K) MA-plots showing average (x-axis) vs difference (y-axis) expression of genes deregulated by siRNA (blue), CRISPR knockout (green) or both siRNA and CRISPR knockout (red) in Dppa2 siRNA (H), Dppa4 siRNA (I), Dppa2 KO (J) or Dppa4 KO (K) cells compared to controls.

**Supplemental Figure 6**

(A) Quantitative RT-PCR analysis for Dppa2 and Dppa4 in wild type (WT, left) and Dppa2/4 DKO (right) ESCs. Cells were transfected with GFP, Dppa2-GFP, Dppa4-GFP or Dppa2-GFP with Dppa4-GFP constructs for 48 hours. Error bars represent average + standard deviation of at least 3 biological replicates. (B) Overexpression of Zscan4c-GFP in wild type (WT, left) and Dppa2^-/-^Dppa4^-/-^ (DKO, right) ESCs. Cells were transfected with GFP or Zscan4c-GFP constructs for 48 hours. Expression of MERVL::tdTomato reporter measured by flow cytometry. Differences are statistically significant (homoscedastic two-tailed t-test, * p-value <0.5, ** p-value <0.01, *** p-value < 0.001, **** p-value < 0.0001). Error bars represent average plus standard deviation of three biological replicates.

**Supplemental Figure 7**

(A-B) Quantitative RT-PCR analysis following transient transfection of untagged Dppa2 and/or Dppa4 in wild type (left) and Dux^-/-^ (right) ESCs, using transfection of an empty vector as a control. (A) Expression of Dux transcript, (B) expression of Dppa2 (green) and Dppa4 (blue) transcripts, Error bars represent standard deviation of 3 biological replicates. EV = empty vector. (C) Wiggle plots showing V5-Dppa2 ChIP seq (top row, green) nad endogenous Dppa4 ChIP-seq in E14 ESCs (third row, blue), P19 embryonal carcinoma cells (fifth row, purple) and Dppa4 overexpression ChIP-seq in 3T3 fibroblast (seventh row, magenta) compared to respective control or input (grey). Data reanalyzed from {Engelen:2015bf, Klein:2018iw} (D-I) Representative wiggle plots showing ChIP-seq binding enrichment for overexpressed Dppa2-V5 (top rows) and endogenous Dppa4 (third, fifth and seventh row) compared to control (second row) or input (fourth, sixth and eighth row). Cell type is denoted on the left and gene structure above. Each figure panel has been scaled independently. Non ZGA genes Syce1 (D) Sohlh2 (E) and Mael (F) show binding enrichment of both Dppa2 and Dppa4 in pluripotent cells. ZGA genes including the Zscan4 cluster (G) Gm428 (H) and Dub1 (I) do not have binding enrichment. (J) quantitative RT-PCR analysis using primers that detect Dux transgene following transfection of GFP or Dux into wild type (WT) or Dppa2^-/-^Dppa4^-/-^ (DKO) ESCs. Bars represent average + standard deviation of three biological replicates.

**SUPPLEMENTAL TABLES**

**Supplemental Table 1:** List of all genes, chromosome coordinates, ENSEMBL gene ID, Description and average log_2_ expression value across the GFP-negative and GFP-positive sorted cells for all the candidate-based screen hits.

**Supplemental Table 2:** List of differentially expressed genes, chromosome coordinates and expression levels in GFP-negative and GFP-positive sorted cells for each of the positive hits from the candidate-based screen.

**Supplemental Table 3:** List of all genes, chromosome coordinates and average log_2_ expression value in control, Dppa2 and Dppa4 siRNA treated ESCs.

**Supplemental Table 4:** List of differentially expressed genes, chromosome coordinates and average log_2_ expression value in control, Dppa2 and Dppa4 siRNA treated ESCs.

**Supplemental Table 5:** List of all genes, chromosome coordinates and average log_2_ expression value in wild type, Dppa2^-/-^, Dppa4^-/-^ and Dppa2^-/-^Dppa4^-/-^ ESCs.

**Supplemental Table 6:** List of differentially expressed genes, chromosome coordinates and average log_2_ expression value in in wild type, Dppa2^-/-^, Dppa4^-/-^ and Dppa2^-/-^Dppa4^-/-^ ESCs.
